# Minimizing treatment-induced emergence of antibiotic resistance in bacterial infections*

**DOI:** 10.1126/science.abg9868

**Published:** 2022-02-24

**Authors:** Mathew Stracy, Olga Snitser, Idan Yelin, Yara Amer, Miriam Parizade, Rachel Katz, Galit Rimler, Tamar Wolf, Esma Herzel, Gideon Koren, Jacob Kuint, Betsy Foxman, Gabriel Chodick, Varda Shalev, Roy Kishony

**Affiliations:** 1 Faculty of Biology, Technion–Israel Institute of Technology, Haifa, Israel; 2 Department of Computer Science, Technion–Israel Institute of Technology, Haifa, Israel; 3 Lorry I. Lokey Interdisciplinary Center for Life Sciences & Engineering, Technion–Israel Institute of Technology, Haifa, Israel; 4 Maccabi MEGA lab, Maccabi Healthcare Services, Tel-Aviv, Israel; 5 Maccabitech, Maccabi Healthcare Services, Tel-Aviv, Israel; 6 Sackler Faculty of Medicine, Tel- Aviv University, Tel-Aviv, Israel; 7 The Department of Biochemistry, University of Oxford, Oxford, UK; 8 Department of Epidemiology, University of Michigan School of Public Health, Ann Arbor, MI, USA

## Abstract

Currently, treatment of bacterial infections focuses on choosing an antibiotic which matches a pathogen’s susceptibility, with less attention to the risk that even susceptibility-matched treatments can fail due to resistance emerging in response to treatment. Here, combining whole-genome sequencing of 1,113 pre- and post- treatment bacterial isolates with machine-learning analysis of 140,349 urinary tract infections (UTIs) and 7,365 wound infections, we find that treatment-induced emergence of resistance could be predicted and minimized at the individual-patient level. Emergence of resistance was common, yet driven not by de novo resistance evolution, but rather by rapid reinfection with a different strain resistant to the prescribed antibiotic. As most infections are seeded from the patient’s own microbiota, these resistance-gaining recurrences can be predicted based on the patient’s past infection history, and their expected risk minimized by machine learning personalized antibiotic recommendations, offering a means to reduce the emergence and spread of resistant pathogens.

Urinary tract infections (UTIs) and wound infections are two of the most common reasons for prescribing antibiotics (1–3). These infections are frequently seeded from bacteria from the patient’s own microbiome: uropathogens can persist for years in the patient’s gut microbiota, which often acts as a reservoir for infection source (4–6). Wound infections are commonly caused by pathogens from the patient’s skin microbiota, as well as pathogens from the gut flora (7). Both UTIs and wound infections can be treated by a range of antibiotics, but resistance is widespread among the causative pathogens and considerable effort is being made to develop strategies to minimize susceptibility mismatches, where an antibiotic is mistakenly prescribed to treat an infection resistant to it (8–10).

Yet, even when an antibiotic is correctly prescribed to treat a pathogen sensitive to it (susceptibility-matched), treatment is a double-edged sword: it may clear the ongoing infection, but it may also select for resistant pathogens among the patient’s resident microbial population, limiting current and future treatment efficacy (11, 12). Indeed, prior antibiotic use is a strong risk factor for resistant UTIs and wound infections at the individual patient level (8, 13–19). This is especially problematic since these infections are often recurrent or chronic, with patients receiving multiple courses of antibiotics (3, 4, 20, 21). Despite the importance of emergence of resistance during or post treatment, we know very little about the mechanisms by which it occurs and we lack strategies to prevent it (22). Currently, antibiotic choice focuses on avoiding antibiotics to which the ongoing infection is already resistant, however, it remains unknown if it is possible to select among the susceptibility-matched antibiotics in ways that minimize the risk of treatment-induced emergence of resistance at the individual-patient level.

Here, to understand and predict personal risk of treatment-induced gain of resistance, we combined whole-genome sequencing of isolates from same-patient recurrent infections with analysis of a longitudinal dataset of UTIs and wound infections collected by Israel’s Maccabi Healthcare Services (MHS) between June 2007 and January 2019. We identified 215,732 MHS patients with at least one record of a UTI (defined as a physician UTI diagnosis followed within 7 days by a positive urine culture with bacterial count >105 cfu/ml; Fig. S2), and 20,373 MHS patients with at least one record of a positive wound infection culture. For these patients, we collected clinical data including antibiotic susceptibilities and species identification from all positive cultures, antibiotic purchases, and patient demographics (age, gender, and pregnancy). For UTI patients we also collected potential comorbidities of chronic kidney disease and diabetes (23), and records of urinary catheterization (24) (Fig. S1, Tables S1-2). Randomly generated patient identifiers were used to link these different patient records. Resistance profiles were classified in accordance with the Clinical and Laboratory Standards Institute (CLSI) guidelines, with intermediate level resistance grouped as sensitive. We identified 41,769 untreated UTI cases (defined as a UTI infection with no antibiotic purchases between 7 days prior to the sample and 28 days following the sample) and 140,349 single-antibiotic treated cases (where, within 4 days of the sample being taken, one of the eight most frequently prescribed systemic antibiotics was purchased: trimethoprim/sulfa, ciprofloxacin, ofloxacin, amoxicillin/CA, cefuroxime axetil, cephalexin, nitrofurantoin, fosfomycin, Table S3). Similarly, for wounds, we identified 7,365 infections treated with one of the five most frequently prescribed oral systemic antibiotics (amoxicillin/CA, ciprofloxacin, cefuroxime/axetil, cephalexin, and trimethoprim/sulfa). We further categorized these infections by their short-term clinical outcomes indicating whether or not they resulted in an “early recurrence”: a second positive sample recorded within 4-28 days following the first sample (13,517 treated UTIs and 7,933 untreated UTIs, 442 treated wound infections).

Even for treatments correctly matching the susceptibility of the infection, early recurrence was common and was associated with infections gaining treatment-specific resistance. Cases were categorized into six groups based on whether their initial infection was sensitive or resistant to the specified antibiotic (S→, R→) and based on their outcome: recurrence with a sensitive or resistant infection, or no recurrence (→S, →R, →⊘) (Fig. 1A). While susceptibility-matched antibiotic treatments (S→) had a lower overall rate of recurrence compared to mismatched treatments (R→), recurrences were still common (UTIs: 9.2%, wound infections: 5.1%) and frequently gained resistance to the prescribed antibiotic (S→R; Fig. 1B,G). Indeed, over 30% of all UTI and 19% of all wound infection recurrences gained resistance following antibiotic treatment (S→R), with this fraction strongly varying by antibiotic, reaching as high as 59% (UTIs) and 27% (wounds) of recurrent infections following treatment with the first-line antibiotic ciprofloxacin (Fig. 1C,H). These gained-resistance cases were strongly associated with treatment: with infections preferentially gaining resistance to the prescribed antibiotic class (Fig. 1F,I), and temporally peaking soon after the last day of the antibiotic course (Figs. 1E, S4). Compared to untreated cases, susceptibility-matched antibiotic treatment had two counteracting effects: while it decreased the overall risk of UTI recurrence (the sum of S→S and S→R), it increased the risk of gained-resistance recurrence (S→R) (Figs. 1D, S5, S6).

The large number of correctly treated infections that subsequently gained resistance could be caused by three possible mechanisms: evolution of resistance through mutations (mutations); through dedicated resistance genes (resistance genes); or through reinfection with a different strain resistant to the antibiotic (strain replacement) (Fig. 2A). To distinguish these possibilities in UTIs, we collected 1,113 isolates from 510 patients who experienced early UTI recurrence during a 4.5 months period (30 November 2017 to 16 April 2018). We focused on Escherichia coli, which accounts for 70 - 95% of all UTIs (Table S4) (4, 22, 25). Sequencing these E. coli isolates, we analyzed the genetic relatedness among same-patient isolates collected before and after treatment and identified any differences in gene content or mutations in antibiotic target and resistance genes (Methods).

The genomic analysis showed that while the same E. coli strain often persists in early UTI recurrences that do not gain resistance, resistance-gaining recurrences were caused by strain replacement. No cases were identified of resistance appearing through point mutations in the originally infecting strain. Analyzing strain relatedness, we find that while reinfection with a new strain was rare in recurrences that did not change resistance to the treatment (19% of S→S, or R→R cases), it was the dominant mode in infections gaining resistance (93% of S→R cases; p = 1x10-27 compared to cases which did not gain resistance, Fisher test, Figs 2B,C, Table S5). For example, despite the ability of E. coli to readily evolve resistance to ciprofloxacin via point mutations in the target enzymes gyrA and parC in lab conditions (26), we found that all UTI cases which gained resistance were caused by reinfection with a different strain, carrying ciprofloxacin-resistant alleles of gyrA and parC (31 of 31 S→R cases, compared to 6 of 25 of S→S cases; p = 4x10-10, Fisher test, Fig. S7) (27). Similarly, while trimethoprim resistance can be acquired via point mutations in the target enzyme dihydrofolate reductase (28), post-treatment resistance was instead conferred by strain replacement (9 of 12 cases), or by the acquisition of a trimethoprim resistant dfrA gene (3 of 12 cases; Table S6) (29). Consistent with untreated cases having a much lower rate of gained resistance recurrence, we found that strain replacement was rare in untreated cases (13%; Fig 2D,E). Furthermore, even for antibiotics for which E. coli resistance is rare, such as fosfomycin and nitrofurantoin (Fig. S8), early recurrence with gained resistance following treatment of an initially sensitive E. coli infection was strongly associated with reinfection with a different resistant strain, yet this time of an entirely different species (Fig. 2F). Overall, 44% of gained-resistance UTI recurrences were caused by a different species (Fig. 2G). A similar pattern was observed for wound infections: while the rate of change of species was low among recurrent wound infections which remained sensitive to the treatment antibiotic (Fig. S9), in most infections which gained resistance (78%), the species which caused the gain of resistance was not present in the original infection (Fig. 2G). Together, these results suggest that selection for existing resistant strains rather than de novo evolution is the predominant mechanism of treatment-induced emergence of resistance.

Since post-treatment resistance was typically caused by strain or species replacement, rather than spontaneous and therefore unpredictable mutations, we asked if emergence of resistance may in fact be predicted at the individual-patient level. As strains are known to recur across same-patient infections even years apart (6), we hypothesized that patients with a history of infections with strains resistant to a given antibiotic to be at higher risk of gained-resistance recurrence following susceptibility-matched treatment with that antibiotic (Fig. 3A). To test this hypothesis, we performed multivariate logistic regressions of the risk of recurrence with gained-resistance given patient demographics and past infection history, among all infections treated with a susceptibility-matched antibiotic (136,047 UTIs, 5,821 wound infections). Despite all of these cases being treated “correctly”, with a susceptibility-matched antibiotic, their risk of recurrence with gained resistance was not uniform: patients with past infections resistant to the currently prescribed antibiotic were at much higher risk of recurring with gained resistance to the treatment compared to patients whose previous infections were sensitive (Fig. 3B,C; see Table S7-8 for regression coefficients). The association between the susceptibility of past infection and the risk of resistance emerging remained significant even for prior infections dating up to 4 years prior to the current UTI (Fig. S10). In contrast, there was no or much weaker association between past infection susceptibility and risk of early recurrence without gain of resistance, showing that this approach specifically predicts the emergence of resistance, rather than merely the risk of early recurrence. Patient’s past infection susceptibility was much more predictive than their past antibiotic purchases, consistent with within-host selection for strains persisting in the microbiome rather than de novo resistance evolution driving treatment-induced gain of resistance (Fig. S11). Finally, beyond the important contribution of personal infection history, we also note the contribution of age and gender to risk of treatment-induced gain of resistance (Table S7-8).

Since some patients were at high risk of their infection gaining resistance to the treated antibiotic, we asked whether the risk of such gained-resistance recurrences may be reduced with an alternative antibiotic. We developed machine learning (ML) algorithms for personalized antibiotic recommendations which minimizes the predicted risk of treatment-associated emergence of resistance for both UTIs and wound infections (Fig. 3D). For each antibiotic, we trained a logistic- regression model to predict the risk of acquiring resistance during or soon after treatment based on patient demographics (age, gender), potential risk factors (pregnancy, catheter use for UTIs), and their record of prior infections including number of past sensitive and resistant isolates. Trained on an initial period and then tested on a temporally separated test-period (UTIs: 14 months; wound infections: 30 months), the models predict the risk of resistance emergence well (the area under the curve ranged from 0.89 for nitrofurantoin to 0.62 for amoxicillin/CA in UTIs, and 0.96 for amoxicillin/CA to 0.58 for cefuroxime in wound infections; ofloxacin was not included since it was not routinely measured during the test period, Fig. S12). More practically, binarizing the patient-specific ML predictions for UTIs into high risk treatments (‘unrecommended’, 15% highest ML predicted risk of gained resistance recurrence), and lower risk treatments (‘recommended’, all others), we found that for every antibiotic, patients for whom the prescribed antibiotic was unrecommended by the ML algorithm acquired antibiotic resistance at a significantly higher rate than those for whom the antibiotic was recommended, even though all of these cases were treated “correctly” with a susceptibility-matched antibiotic (Fig. 3E; the trends are robust with respect to the recommendation threshold; Fig. S13).

Analyzing all susceptibility-matched treated cases in the test-period, we found that in most cases there was an alternative susceptibility-matched antibiotic which had a lower patient-specific predicted risk of resistance emerging compared to the antibiotic prescribed by the physician (77% of UTIs; 76% of wound infections). Choosing for each patient the antibiotic with the minimal ML predicted risk of emergence of resistance (ML-recommended) reduces the overall risk of emergence of resistance by 70% for UTIs and 74% for wound infections compared to the risk for physician-prescribed treatments (Fig. 3F,G). Since many factors contribute to the rate at which physicians prescribe each antibiotic, such as antibiotic efficacy, cost, ease of use and side effects, we also developed a constrained antibiotic recommendation model that minimizes the risk of emergence of resistance while preserving the same prescription frequency of each antibiotic as prescribed by physicians during the test period (Fig. S14) (14). Even these constrained antibiotic recommendations, which merely permute the physician prescribed antibiotics among patients, can reduce the risk of resistance emerging after treatment by 48% for both UTIs and wound infections compared to the physician-prescribed antibiotics (Fig. 3F,G). To demonstrate that these constrained recommendations could be made on a case-by-case basis, we also show that the model remains effective when constrained to the physician prescription frequency during a temporally separated period prior to the final model evaluation period (Fig. S14). We note that a simpler algorithm that randomly chooses an antibiotic but avoids antibiotics to which the patient had past resistance can still reduce the risk of resistance emerging after treatment, albeit at a lower frequency than either of the ML models, consistent with the contribution of other factors including age, gender and the more quantitative representation of past infections (Fig. 3F,G). Furthermore, analyzing the distribution of ML-recommended antibiotic for subsets of patients, such as those with past resistance to a specific antibiotic, may help guide treatment recommendations more broadly (Fig. S15). Importantly, the constrained ML models also reduce overall predicted risk of early recurrence (the sum of S→S and S→R) showing that this personalized approach not only reduces gained-resistance recurrences, but by doing so may also reduce the overall recurrence risk (Fig. S17).

In conclusion, while much effort is invested in methodologies for matching antibiotic treatment to infection susceptibility, susceptibility-matched treatments often fail as they select for emergence of resistance via reinfection with different strains specifically resistant to treatment. The strong association between such treatment-induced selection for resistance and personal history of past resistant infections suggests a patient-specific strain reservoir. Given the known role that uropathogens and wound pathogens persisting in the patient’s microbiome have in seeding new infections (4–6, 31, 32) and the collateral effect that antibiotics can have on the patient’s microbiome (33–35), it will be interesting to see whether these emerging resistant strains can be detected in the patient’s fecal or skin flora. Regardless of the exact source of these reinfecting resistant strains, our results show that a patient’s past infection susceptibility data and patient demographics can be used to predict early recurrence with gained resistance following susceptibility-matched antibiotic treatment. We hope these results will serve as a basis for a personalized treatment approach that minimizes the selection and spread of resistant pathogens at both the individual patient and population levels.

## Supplementary Material

Supplementary Materials

## Figures and Tables

**Figure 1:**
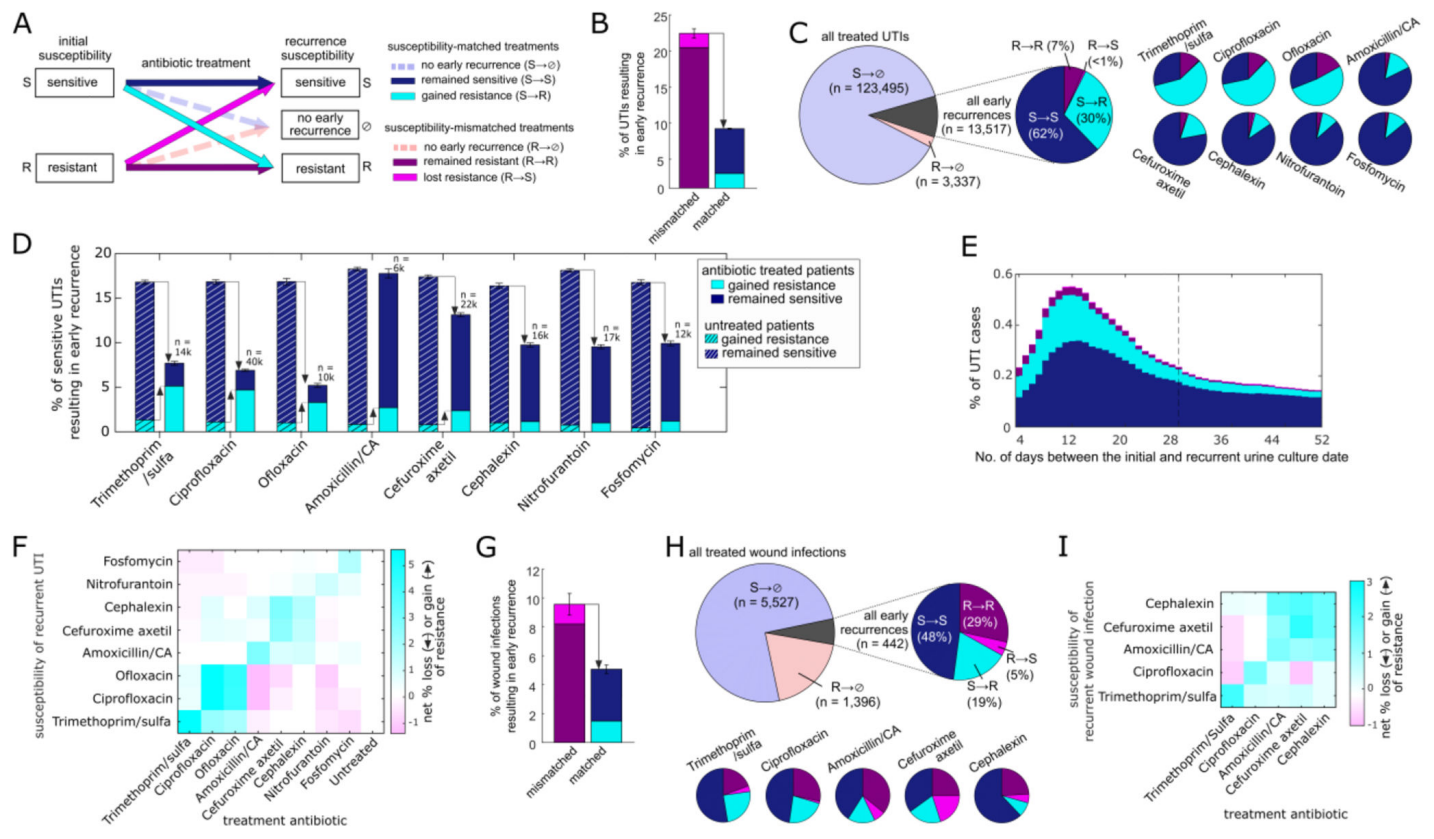
Post-treatment recurrences are strongly associated with the infection gaining resistance specifically to the treated antibiotic. (A) Each infection case was categorized into one of six possible outcomes based on the susceptibility and treatment outcome. (B,G) The overall rate of recurrence for UTIs (B) and wound infections (G) following either susceptibility-matched or susceptibility-mismatched antibiotic treatments. (C,H) The percentage of all antibiotic treated UTIs (C) and wound infections (H) resulting in early recurrence, and a breakdown of these early recurrences by their pre- and post- treatment susceptibility to the treatment antibiotic, for all treated cases and for each of the most frequently prescribed antibiotics. (D) The rate of early recurrence for UTIs initially sensitive to the specific antibiotic and either treated with this antibiotic (solid bars) or untreated (hashed bars). The cases are further categorized based on whether they recurred still sensitive to the specified antibiotic (blue) or recurred while gaining resistance to it (cyan). Susceptibility-matched treatment decreases the overall risk of early recurrences (down-pointing arrow), yet increases the risk of recurrence with gained resistance (up-pointing arrows). (E) The rate of UTI recurrences occurring on each day following antibiotic treatment (7-day moving average). Each recurrent case is categorized by pre- and post- treatment susceptibility to the prescribed antibiotic as shown in panel A. The dashed vertical line shows the 28-day threshold used to define early recurrences. (F,I) The net change in susceptibility of early recurrent UTIs (F) and wound infections (I). For infections treated with each antibiotic (x-axis) or untreated (UTIs), the percentage of gain of resistance (cyan) minus loss of resistance (magenta) to each specified antibiotic is shown (y-axis).

**Figure. 2:**
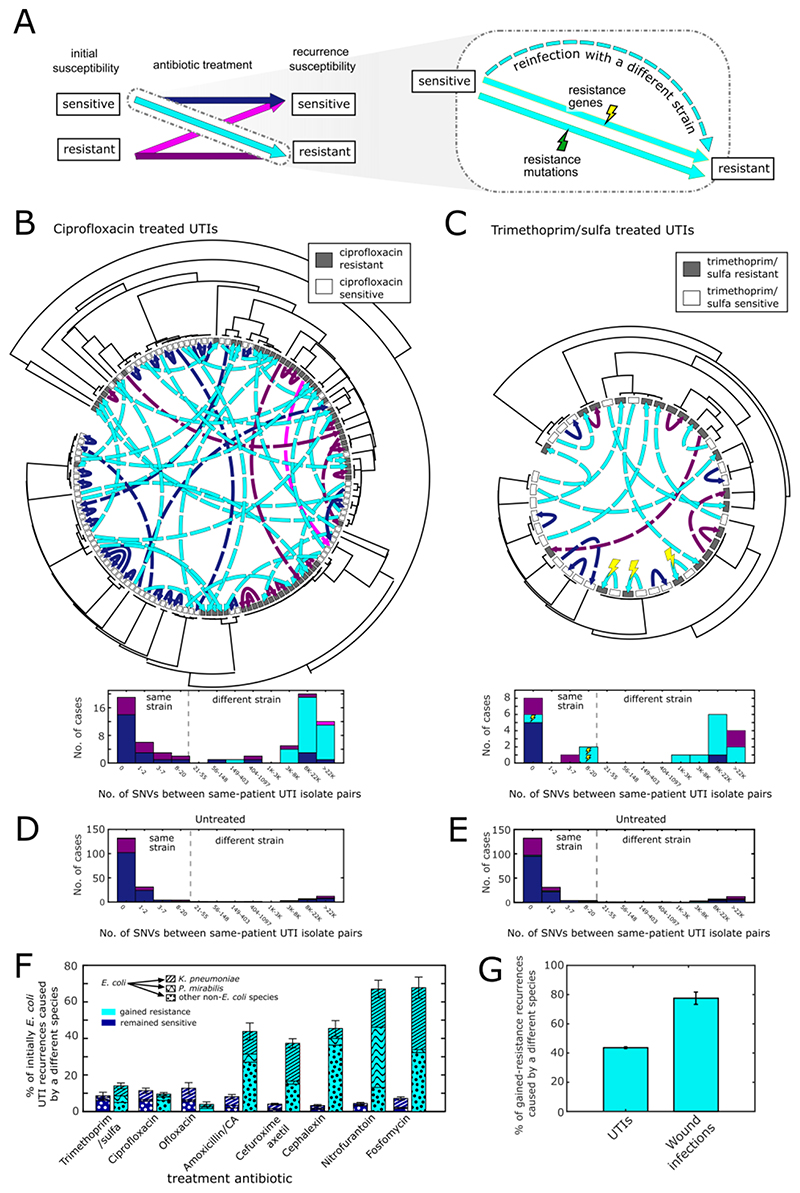
Genomic analysis of infecting pathogens before and after antibiotic treatment. (A) Infections which recurred with gained resistance following treatment (cyan) could be a consequence of acquiring resistance-conferring mutations (green lightning), resistance conferring genes (yellow lightning), or reinfection with a different strain resistant to the antibiotic (dashed arrow). (B,C) Phylogenetic trees of E. coli urine culture isolates collected from patients who experienced early recurrence following treatment with ciprofloxacin (B) or trimethoprim/sulfa (C), with isolate resistance/sensitivity to the prescribed antibiotic indicated by grey/white boxes. Same-patient isolates are connected with arrows whose color and style represent change in infection susceptibility and mechanism of gain of resistance (as defined in panel A). Histograms show the genetic distance, in number of single nucleotide variations (SNVs), between these same patient isolate pairs, again categorized by infection susceptibility and mechanism of gain of resistance (as defined in panel A). Vertical dashed lines represent the threshold used to define same-strain versus different-strain recurrences. (D,E) Histograms of the genetic distance in SNVs between same-patient isolates in untreated cases categorized by infection susceptibility to ciprofloxacin (D) or trimethoprim (E). (F) The percentage of E. coli infections treated with a susceptibility-matched antibiotic which resulted in early recurrence with different non-E. coli species (bar patterns), for recurrences which remained sensitive (blue) or gained resistance (cyan) to the prescribed antibiotic. (G) The percentage of gained-resistance recurrences in all UTIs and wound infections which were caused by reinfection with a different species.

**Figure 3:**
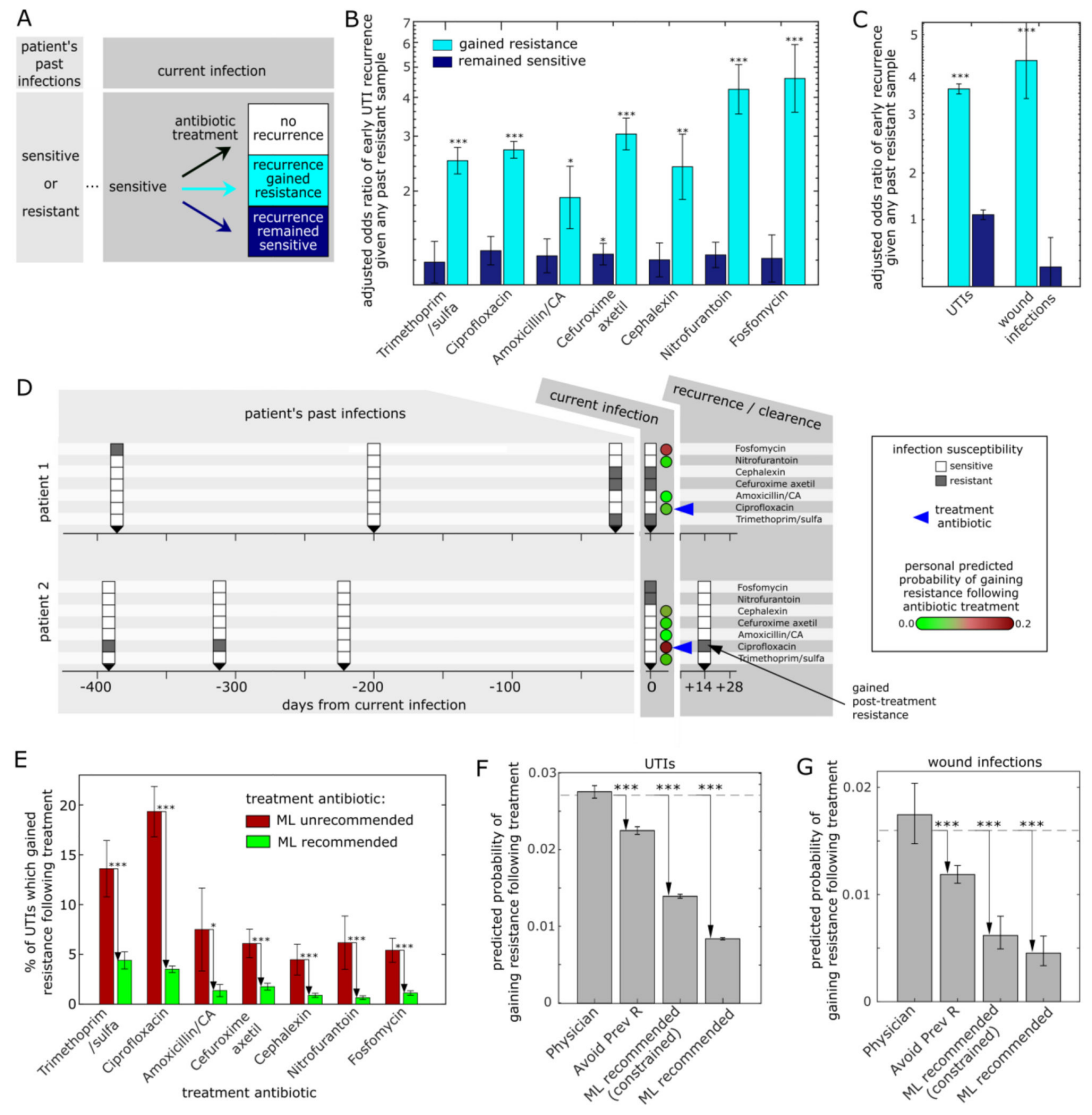
Personalized, antibiotic-specific, predictions of treatment-induced emergence of antibiotic resistance. (A) Schematic showing the possible outcomes of susceptibility-matched antibiotic treatment for patients with a recorded history of prior infection susceptibility to the currently prescribed antibiotic. (B) Odds ratio of risk of early recurrence which gained resistance (cyan) or remained sensitive (blue) given the patient’s prior history of resistant infections (binary 1/0: any prior resistance to the prescribed antibiotic, or no prior resistance to the prescribed antibiotic). For each antibiotic, all susceptibility-matched treated cases for patients with any prior infections within the past 3 years are considered. Odds ratios are adjusted for demographics (age, gender) and potential risk factors (pregnancy, catheter use). (C) The adjusted odds ratio of early recurrence given the patient’s prior history of resistant infections for all antibiotic treatments combined for both UTIs and wound infections. (D) Timeline of two example patients showing, the susceptibilities of their current (t=0) and prior (t<0) infections for each antibiotic (white/grey for sensitive/resistance), as well as their ML predicted probability of recurrence with gained resistance upon treatment of their current infection with each of the antibiotics (circles, green-to-red colormap). Despite both patients treated with the same antibiotic for which their infection was sensitive, ciprofloxacin (blue arrow), they had very different ML personal predicted risk of gaining post-treatment ciprofloxacin resistance and indeed varied accordingly in the treatment outcome. (E) The percentage of UTIs within the 14-month test period which gained resistance following treatment for cases prescribed an antibiotic that was unrecommended (red, 15% highest predicted risk) or recommended (green, 85% lowest predicted risk) by the ML algorithm (these results are robust to choice of grouping intermediate level resistance with resistant, Fig. S16). (F,G) The overall predicted probability of gaining resistance for all UTIs (F) and wounds (G) during the test period for 4 different antibiotic prescription methods: the actual antibiotic prescribed by the physician; an algorithm that randomly chooses an antibiotic but avoids antibiotics to which the patient had past resistance, and the ML recommendation either unconstrained, or constrained such that each antibiotic is recommended at the exact same frequencies as prescribed by the physicians. The dashed line represents the actual gained-resistance rate for the physician-prescribed antibiotics during the test period. * p < 0.05; ** p < 0.005; *** p < 0.0005.

## Data Availability

The clinical data that support the findings of this study are available from Maccabi Healthcare Services but restrictions apply to the availability of these data, which were used under license for the current study, and so are not publicly available. Access to the data is, however, available upon reasonable request and signing an MTA agreement with Maccabi Healthcare Services. Analysis code is available from https://github.com/Technion-Kishony-lab/Antibiotic-treatment-failure. All urine culture isolate whole-genome sequencing data generated in this study have been deposited in the SRA database and are available here: https://www.ncbi.nlm.nih.gov/sra/PRJNA786867. Treatment and susceptibility data for the sequenced isolates are provided are provided with this paper.
