# Supplementary material for: Minimizing treatment-induced emergence of antibiotic resistance in bacterial infections
Source: Science. Author manuscript; Available in PMC 2022 Mar 4. (PMC7612469; doi:10.1126/science.abg9868)
Supplement: Supplementary Materials [file EMS143775-supplement-Supplementary_Materials.pdf]

Science
AAAS

Supplementary Materials for

Minimizing treatment-induced emergence of antibiotic resistance in bacterial infections

Mathew Stracy, Olga Snitser, Idan Yelin, Yara Amer, Miriam Parizade, , Rachel Katz, Galit Rimler, Tamar Wolf, Esma Herzel, Gideon Koren, Jacob Kuint, Betsy Foxman, Gabriel Chodick, Varda Shalev, Roy Kishony.

Correspondence to: rkishony@technion.ac.il.

This PDF file includes:

Materials and Methods
Supplementary Text
Figs. S1 to S17
Table S1 to S8
References (36-38)

Materials and Methods

Data collection

Records from patients with UTIs or wound infections were collected between June 2007 and January 2019 by Maccabi Healthcare Services (MHS), the second largest Health Maintenance Organization in Israel. We identified 352,665 MHS patients who had at least one positive urine culture during this period (with a bacterial count >10^5 cfu/ml) referred from one of over 100 community clinics located nationwide. We identified 17,735 MHS patients with at least one record of a positive wound infection culture. For each of these patients, we extracted the following information:

Positive culture reports: Each report included: 1) Unique patient identifier; 2) the date the sample was taken; 3) List of isolates cultured with species identification (for UTIs 96.4% of samples had one isolate and 3.6% of samples had more than one isolate; for wound infections 63.8% of samples had one isolate and 36.2% of samples had more than one isolate); 4) Antibiotic susceptibility profile of the isolates measured using a VITEK 2 system and classified in accordance with The Clinical and Laboratory Standards Institute (CLSI) guidelines into Sensitive, Intermediate and Resistant for each antibiotic tested; 5) Pregnancy flag indicating the patient was pregnant when the sample was taken.

Demographics: We identified and retrieved the demographics of all patients with culture reports. For each patient this included 1) the date of birth used to calculate the patients age at the time the sample was collected; 2) Gender.

Antibiotic purchase reports: All antibiotic purchases by prescription are routinely recorded in MHS databases. We identified and retrieved all antibiotic purchases made by patients with culture reports (total of 6,690,425 antibiotic purchases for UTI patients over 11.5 years; total of 604,902 antibiotic purchases for wound infection patients over 11.5 years;). Internal MHS drug codes were converted to ATC classifications of antibiotics. Each purchase record included: 1) Unique patient identifier; 2) Internal MHS product code, which was translated to an ATC drug code, 3) Date of purchase.

For UTI patients we further extracted the following information:

Diagnosis: To confirm that patients with bacteriuria identified from the culture reports had symptomatic UTIs we also retrieved physician diagnoses of UTIs for all patients with positive urine culture reports. For each patient this consisted 1) Unique patient identifier to be linked to the identifier of the culture record; 2) Date of the diagnosis. UTI cases were defined as those with a coinciding positive culture report and UTI diagnosis within 7 days of each other. The date of the UTI was defined by the date the urine culture was taken.

Patient comorbidities: We retrieved the diagnosis of potential comorbidities of chronic kidney disease and diabetes for all 352,665 patients with culture reports. For each patient this included 1) date of diagnosis; 2) Disease diagnosed (chronic kidney disease or diabetes). We identified all patients who had received a diagnosis prior to the recorded UTI.

Catheter use: We retrieved the records of all urinary catheterization procedures for all patients with culture reports, including the date of the procedure. We identified patients who had ever had any of these procedures prior to he recorded UTI. We separately also identified patients who had had a catheterization procedure within the 30 days preceding the UTI.

Randomly generated unique patient identifiers were used to link these different data sources.

Study design and exclusion criteria

A UTI case was defined as a positive urine culture and a physician diagnosis of UTI within 7 days of each other. Potentially asymptomatic bacteriuria cases of a positive culture, but without an associated physician UTI diagnosis were excluded (see flow diagram Fig. S1). UTI cultures were taken for 49% of the recorded UTI diagnoses, with a positive rate of 66% (Fig. S2). Since pyelonephritis and cystitis differ in their modes of recurrence (31), we further excluded cases associated with pyelonephritis diagnosis (Fig. S1). A wound infection case was defined as any positive wound swab culture. For both wound infections and UTIs, analysis was performed at the level of cases; patients with more than one case could enroll more than once.

We included in our analysis cases treated with one of the most frequently prescribed systemic antibiotics for each infection (UTIs: trimethoprim/sulfa, ciprofloxacin, ofloxacin, amoxicillin/CA, cefuroxime axetil, cephalexin, nitrofurantoin, fosfomycin; wound infections: amoxicillin/CA, cefuroxime axetil, cephalexin, trimethoprim/sulfa, ciprofloxacin). All cases treated with a topical antibiotic only were excluded from the analysis (Fig. S1B). Antibiotic treated cases were defined as infection cases treated with a single antibiotic (UTIs: had exactly one antibiotic purchase within 4 days of the sample being taken: 140,349 cases; Wound infections: had one antibiotic purchase within 7 days of the sample being taken: 7,365 cases). Susceptibility to these antibiotics was routinely measured for cultured isolates throughout the study period, with the exception of ofloxacin which was not measured from 2014 onwards and cephalexin which was only measured starting from 2014. For Staphylococcus aureus isolates oxacillin resistance was routinely measured to identify MRSA and was used to determine β-lactam resistance according to CLSI guidelines in cases where the relevant prescribed antibiotic was not measured. For each infection, we defined the susceptibility to each of the antibiotics as sensitive if all isolates measured from the sample were sensitive or intermediate and resistant if any isolate from the same sample were resistant. The susceptibility was defined as not available if the antibiotic was not measured. Cases treated with an antibiotic other than one of the specific antibiotics being studied, cases with more than one antibiotic purchased, and cases with an antibiotic purchase outside the 4-day or 7-day cut-off were excluded (cases not fulfilling the antibiotic treated criteria, Fig. S1). Cases to which the relevant susceptibility was not measured were also excluded (Fig. S1). We also analyzed untreated UTI cases, defined as UTI case with no antibiotic purchases during the 7 days prior to the sample, and no antibiotic purchases during the following 28 days or before the next sample (whichever comes first) (41,769 cases). Patient demographics for the treated and untreated UTI cases are shown in Tables S1. Since wound infections consist of a broader variety of infections compared to UTIs we did not compare the rate of recurrence between treated and untreated cases. Patient demographics for the treated wound infections are shown in Table S2.

Case outcomes and susceptibility categorization

For each infection case, we labelled two key outcomes: 'early recurrence' and 'gain of resistance'. Early recurrence was defined as cases followed by a second positive sample within 4-28 days following the first sample (13,517 and 442 antibiotic treated early recurrent UTI and wound infection cases, respectively). Cases that recurred in <4 days were excluded in this analysis. The majority (55% UTIs; 57% wound infections) of the antibiotic treated cases resulting in early recurrences also had a second treatment (antibiotic purchase within 4 or 7 days of the recurrence for UTIs and wounds, respectively), suggesting the recurrences were symptomatic infections. Error bars for rates of early recurrence represent the Binomial standard error.

Infection cases were further categorized into 6 groups based on whether their initial infection was sensitive or resistant to the specified antibiotic (S→, R→) and based on their outcome: early recurrence with a sensitive or resistant infection, or no early recurrence (→S, →R, →∅). Gain of resistance was defined as an initially sensitive case resulting in early recurrence resistant to the specified antibiotic (S→R). Antibiotic treated cases which were initially sensitive to the prescribed antibiotic were defined as 'susceptibility-matched' and those that were initially resistant were described as 'susceptibility-mismatched'.

Multivariate logistic regressions

The outcome of each susceptibility-matched infection (total: 136,047 UTI cases; 4,731 wound infection cases) was labelled as gained resistance (1, early recurrences which gained resistance) or not (0, no early recurrence, or early recurrence which remained sensitive). As features, we considered: X_m^{Gender}, 0/1 for males/females; $X_m^{Pregnancy}$, 0/1 indicating pregnancy; X_m^{Age}, 0/1 indicating patient age at the time of UTI sampling in groups of 10 years (0–10,11– 20,…,91–100 years), Sample history: For a given sample, we consider all earlier samples of the same patient within the past 3 years. X_{mk}^{Prev}, 0/1, indicating if the patient had any previous samples resistant to antibiotic k. For UTIs we also considered $X_m^{Catheter}$, 0/1 indicating any prior catheter use; The regression model for panels Fig. 3B,C included the following terms:

$$ln\left(\frac{P_k^m}{1-P_k^m}\right) = C_k^{Const} + C_k^{Gender}X_m^{Gender} + C_k^{Pregnancy}X_m^{Pregnancy} + \sum_{j=2}^{10} C_{kj}^{Age}X_{m,j}^{Age} + C_k^{Prev}X_{mk}^{Prev}$$

With $C_k^{Catheter}X_m^{Catheter}$ also included as a term for UTIs. Logistic regressions were performed in Matlab using the glmfit function for cases treated with each antibiotic $k = 1,2,\ldots,7$ where the antibiotic was susceptibility-matched to the infection. Only cases with at least one previous sample with susceptibility measurements to antibiotic k in the past 3 years are considered in each regression. All such cases over the entire 11.5-year dataset were included. As a control, we also performed the regression with the same features but with the outcome of 0/1 indicating early recurrences which remained sensitive. Logistic regressions were performed in Matlab using the glmfit function. Regression coefficients for Fig. 3B,C are shown in Table. S7, S8.

For personalized antibiotic recommendations (Fig. 3D, E), the following parameters were included: $X_{mk}^{PrevR}, 1,2, \dots, N_{Res}$ where N_{Res} is the maximum number of previous samples in the past 3 years which were resistant to antibiotic k, and $X_{mk}^{PrevS},\ 1,2, \dots, N_{Sen}$, where N_{Sen} is the maximum number of previous samples sensitive to antibiotic k. The regression models were trained on all cases during the 10-year 'training period' which were treated with each antibiotic $k = 1,2, \dots, n$ where the antibiotic was susceptibility-matched to the infection (including patients with no prior infections, in addition to those with prior infections). The regressions included the following terms:

$$ln\left(\frac{P_k^m}{1-P_k^m}\right) = C_k^{Const} + C_k^{Gender} X_m^{Gender} + C_k^{Pregnancy} X_m^{Pregnancy} + \sum_{j=2}^{10} C_{kj}^{Age} X_{m,j}^{Age} + C_k^{PrevR} X_{mk}^{PrevR} + C_k^{PrevS} X_{mk}^{PrevS}$$

With $C_k^{Catheter} X_m^{Catheter}$ also included as a term for UTIs. Ofloxacin was excluded from the drug recommendations for UTIs since its susceptibility was not routinely measured during the test period. For each of the antibiotics, $k = 1,2, \dots, n$ the probability of resistance emerging following treatment was fit to the Y_k^m indicating if the case did or did not result in early recurrence which gained resistance to the treatment antibiotic k. For each regression any parameters with insufficient data (for binary parameters where the total cases of 0 < 15 or cases of 1 < 15) were excluded.

For the regression models comparing treated and untreated UTIs in Fig. S4, the demographics adjusted risk of recurrence in treated compared to untreated cases we defined the Treatment parameter: For each case, we defined if it was treated with antibiotic k or was untreated $X_{mk}^{Treated}$: 0/1, for untreated/treated. The regression included the following terms:

$$ln\left(\frac{P_k^m}{1-P_k^m}\right) = C_k^{Const} + C_k^{Gender} X_m^{Gender} + C_k^{Pregnancy} X_m^{Pregnancy}$$

$$+ C_k^{Catheter} X_m^{Catheter} + \sum_{j=2}^{10} C_{kj}^{Age} X_{m,j}^{Age} + C_k^{Treated} X_{mk}^{Treated}$$

For each of the antibiotics, $k = 1,2, \dots, 7$ the probability of resistance emerging following treatment was fit to Y_k^m indicating if the case did or did not result in early recurrence (4-28 days after the first sample) which gained resistance to the antibiotic k. For each antibiotic k all cases initially sensitive to k either treated with antibiotic k or untreated over the entire 11.5-year dataset were included. This regression was repeated as described above for the probability of any early recurrence (i.e. Y_k^m indicated if the case resulted in any early recurrence 4-28 days after the first sample, including those which remained sensitive and those which gained resistance). For susceptibility-mismatched cases, the regression was performed for each case initially resistant to antibiotic k and either treated with antibiotic k or untreated.

<u>Algorithm for patient-specific antibiotic recommendations</u>

A logistic regression model as described above was used to determine the antibiotic, $k = 1,2, \dots, n$, with the lowest risk of resulting in early recurrence with gained resistance following

each susceptibility-matched treated case (ofloxacin was excluded from the UTI antibiotic recommendations since its susceptibility was not routinely measured during the test period). The regression was performed on all cases sensitive to antibiotic k and treated with antibiotic k occurring between June 2007 to October 2017 for UTIs (training period; total: 113,718 cases) or June 2007 to May 2016 for wound infections (training period; total: 3,028 cases). The regression included the patient's demographics (as above) and the number of prior infections resistant/sensitive to antibiotic k during the past 3 years. This model was tested on all susceptibility-matched cases (total: 12,765 UTI cases, 1,703 wound infection cases) which occurred within a temporally separated test period (November 2017 to January 2019 UTIs, June 2016 to January 2019 UTIs,). For each such case m in the testing period, the probabilities of resistance emerging following treatment, P_k^m, was calculated for each antibiotic k based on the trained logistic regression models. The algorithm recommends the antibiotic K_{rec}^m which matched the susceptibility of the infection and had the lowest predicted probability of emergence of resistance: $K_{rec}^m = argmin_k(P_k^m)$.

Constrained (cost-adjusted) algorithm for antibiotic recommendations

In the constrained ML model, P_k^m was first cost-adjusted as described by Yelin *et al* (14) such that the total number of each antibiotics recommended equals the total number prescribed by physicians in the dataset. Briefly: the constrained algorithm takes the probabilities from the unconstrained ML model, P_k^m for all cases, *m*, and each of the seven antibiotics, *k*. As for the unconstrained algorithm all the susceptibility mismatched antibiotics for each case are excluded. The algorithm also takes the target total number of prescriptions of each antibiotic during the test period n_k^{target} (with $\sum_{k=1}^{N_{Cases}} n_k^{target} = N_{Cases}$). The algorithm returns the optimal recommended antibiotic treatments for each case such that the overall expected rate of resistance emerging is minimized while the overall number of recommended antibiotics satisfies $n_k = n_k^{target}$.

Algorithm for antibiotic recommendations which avoids previous resistance.

To compare the complete ML algorithm, which also include patient demographic information in addition to their past sample susceptibility data, we also created a simple algorithm which avoids prescribing antibiotics to which the patient has any record of prior resistance. In cases where the physician prescribed antibiotic was one to which the patient has a record of prior resistance over the past 3 years an antibiotic was randomly assigned from among the alternative susceptibility-matched antibiotics to which the patient did not have a record of past resistance. The algorithm retained the physician antibiotic choice in cases where the patient did not have a record of prior resistance to that antibiotic.

Comparison of physician prescribed ML recommended and ML not recommended antibiotics

For each antibiotic $k = 1,2,...,n$ a threshold was determined based on the 85th percentile of the probabilities of resistance emerging following treatment, P_k^m, for all susceptibility-matched treated case in the 14-month testing period, $m = 1,2,..,N_{samples}$. Each case was then categorized based on if the P_k^m for the antibiotic prescribed by the physician was above (i.e. within the 15% of highest P_k^m values) or below (i.e. within the 85% of lowest P_k^m values) this threshold and labeled as not-recommended and recommended, respectively. The percentage of cases in which resistance emerged following treatment was determined in both categories for each antibiotic. Error bars

represent the Binomial standard error. The significance between the rate of resistance emerging for the recommended and not-recommended categories was determined with a Fisher's exact test.

Statistical significance of predicted risk of resistance emerging

To determine the statistical significance of the ML predicted risk of resistance emerging following susceptibility-matched treatment we performed 10,000 bootstrapping simulations in which we randomly sampled with replacement all 12,765 P_k^m (UTIs) or all 1,703 P_k^m (wound infections) for each of the 4 scenarios over the test data set (a random antibiotic k for each case; the physician prescribed antibiotic for each case; the antibiotic recommended by the unconstrained ML model for each case; the antibiotic recommended by the constrained ML model for each case). For each of these scenarios, we report the 95% confidence interval of across the 10000 bootstrapped simulations from the 2.5th and 97.5th percentiles. To estimate the significance between the models, we calculated the error function based on the average and standard deviation of the difference between the probabilities from the two models across the 10,000 bootstrapping simulations.

UTI isolate collection protocol

For genetic analysis, we collected and performed whole-genome sequencing of urine culture isolates from UTI cases with early recurrence. Isolates from midstream urine cultures were collected by MHS over a 4.5 months (30 November 2017 to 16 April 2018). We focused on cases with an *E. coli* infection, representing 73% of all single species infections. After standard microbiological and susceptibility analysis the isolates were stored on a rolling 42-day basis (to allow collection of more isolates during the collection period, the range of recurrence days was extended to 42 days for the genetic analysis). Patients who returned with a positive second sample within 42 days of the first sample were identified and the isolate plates from both cultures were sent to the Technion where they were cultured in MacConkey media overnight and 50% glycerol added for storage at -80°C. A total of total of 1,113 isolates from 510 patients were collected and successfully sequenced (437 patients with two samples and 73 patients with 3-6 samples). All cases fulfilling these criteria were designated for collection regardless of demographics, resulting in an unbiased sampling of the pool of early recurrent cases (Table S1).

Whole genome sequencing and genomic analysis of urine culture isolates

Isolates were prepared for sequencing following the protocols described in Baym et al (36). For each isolate 80 μl of thawed culture was inoculated into 800 μl of LB broth in a 96 deep-well plate and incubated overnight at 37°C. DNA was extracted using a commercial kit (Macherey-Nagel, NucleoSpin 96 Tissue) and Nextera sequencing libraries were prepared. Sequencing was performed on an Illumina HiSeq 2500 machine in rapid mode to produce 125 base paired-end reads. Reads were trimmed to remove contamination by the Nextera adapter and filtered to remove reads with low-quality bases (>2 bases with a Phred Score of <20), yielding an average of 3.78 M reads per sample (s.d. = 1.4 x10^6).

A preliminary phylogenetic tree was constructed using k-mers of unaligned trimmed and filtered reads using MASH software (37). 20 isolates were picked representing all clades in this preliminary tree. 10,000 reads from each of these isolates were blasted against the NCBI Enterobacteria database to identify reference genomes with maximum overall hits. For each isolate reads were aligned to this reference genome using Bowtie 1.2.1.1, allowing a maximum of 3

mismatches per read. Unaligned reads were subsequently aligned to the next best genome for a total of 20 genomes. Only chromosomal reference genomes were picked to ensure the genetic distance used to categorize isolates into the same or a different strain was based on chromosomal differences not plasmid diversity. Base-pair calls were identified using SAMtools and BCFtools 0.1.19. Categorization of isolates into the same or a different strain was done based on a threshold of 20 single nucleotide variants (SNVs) identified within the shared chromosome between the same-patient isolate pairs. Phylogenetic trees were determined based on the pairwise SNVs identified within the shared chromosome of all isolates included in the tree. A chromosome position was identified as a SNV if more than a single allele was identified across isolates using a quality threshold of $FQ < -80$. To verify that same-patient isolate pairs categorized as the same strain had the same multi locus sequence type (MLST) we used Staramr software to identify the MLST for each isolate. For same-patient isolate pairs identified as the same strain (less than 20 SNVs difference) with an MLST identified for both isolates 100% were the same MLST type. In contrast, for same-patient isolate pairs categorized as a different strain (20 or more SNVs difference) only 6% (8 out of 126 pairs) were the same MLST.

The presence of known antibiotic resistance genes were identified using ResFinder (version 3.2) and fluoroquinolone-resistance conferring SNPs were identified with PointFinder (27) both using databases retrieved on 2019-07-01. For this analysis a *de novo* assembly was constructed using Spades (version 3.13.2) (38) using trimmed and quality filtered paired-end reads to include both chromosomal and any plasmid sequences for each isolate which was used as input for the ResFinder and PointFinder software.

<u>Study oversight</u>
The study protocol was approved by the ethics committee of Assuta Medical Center, Tel-Aviv, Israel, which did not require that patients provide written informed consent.

Supplementary Text

<u>Genetic determinants of resistance in UTIs which gained resistance</u>
Of the 31 sequenced cases which acquired ciprofloxacin resistance following ciprofloxacin treatment, all 31 were caused by a different strain as defined by >20 SNVs between the pre- and post-treatment isolates (Table S5). In 30 out of 31 of the newly infecting ciprofloxacin resistant strains we identified at least 3 known fluoroquinolone resistance conferring point mutations in the target topoisomerase genes gyrA, parC, parE (Fig. S7) were identified by the Pointfinder software. On average each of these post-treatment isolates had 3.9 [95% CI 3.5 - 4.2] known fluoroquinolone resistance conferring SNPs compared to 0.4 [CI 0.2 - 0.6] in the original sensitive strains. The mutations identified are in the pre- and post-treatment isolates are shown in Fig. S6. In one case we did not identify any known fluoroquinolone resistance conferring point mutations in the ciprofloxacin resistant post-treatment isolate, however we identified the gene qnrB28 which is known to confer fluoroquinolone resistance (Table S4).

Of the 12 cases which acquired trimethoprim/sulfa resistance following trimethoprim/sulfa treatment, 9 were caused by a different strain as defined by >20 SNVs between the pre- and post-treatment isolates, and 3 were caused by the same strain (Table S5). In the three cases which were the same strain but which acquired post-treatment resistance, we identified the trimethoprim

resistance gene dfrA17 in the post-treatment isolates and identified no trimethoprim resistance gene in the pre-treatment isolate (Table S6). In all 9 cases caused by a different strain a dfrA gene was identified in the post-treatment isolate.

All 6 cases which acquired cefuroxime resistance following cefuroxime treatment were caused by a different strain as defined by >20 SNVs between the pre- and post-treatment isolates. In 6 of the post treatment isolates a blaCTX extended-spectrum β-lactamase (ESBL) gene was identified, and in the remaining case a blaSHV ESBL gene was identified (Table S6).

Association of past UTI susceptibility with risk or emergence of resistance for initially *E. coli* and non-*E. coli* infections

Genomic analysis of *E. coli* infections showed that strain replacement was the main cause of early recurrence with gained resistance (Fig. 2). This provided the motivation for the predictive models for the risk of gaining resistance based on patient demographics and past resistant infections. Although *E. coli* infections account for the majority of UTIs, it is possible that infections with other species gain resistance predominantly via other mechanisms. We therefore tested if the associations between past UTI susceptibility with risk or emergence of resistance shown in Fig. 3B (with all species included), remained significant when only initially *E. coli* cases or only initially non-coli cases were considered (Fig. S11).

Intermediate resistance grouping

Since the rates of intermediate resistance was low for most antibiotics (Fig. S7; ciprofloxacin 0.4%, ofloxacin 0.4%, Trimethoprim/sulfa 0.0%, and fosfomycin 0.0%) and intermediate levels of resistance do not exclude prescription of an antibiotic, we grouped intermediate resistance with sensitive in our analysis. To verify that the association of past UTI susceptibility with risk or emergence of resistance remained significant regardless of the choice of intermediate grouping, we performed the same analysis as described in the main text, with intermediate categorized with resistant instead of sensitive (Fig S16), showing that the choice of grouping makes minimal difference to the predictive models.

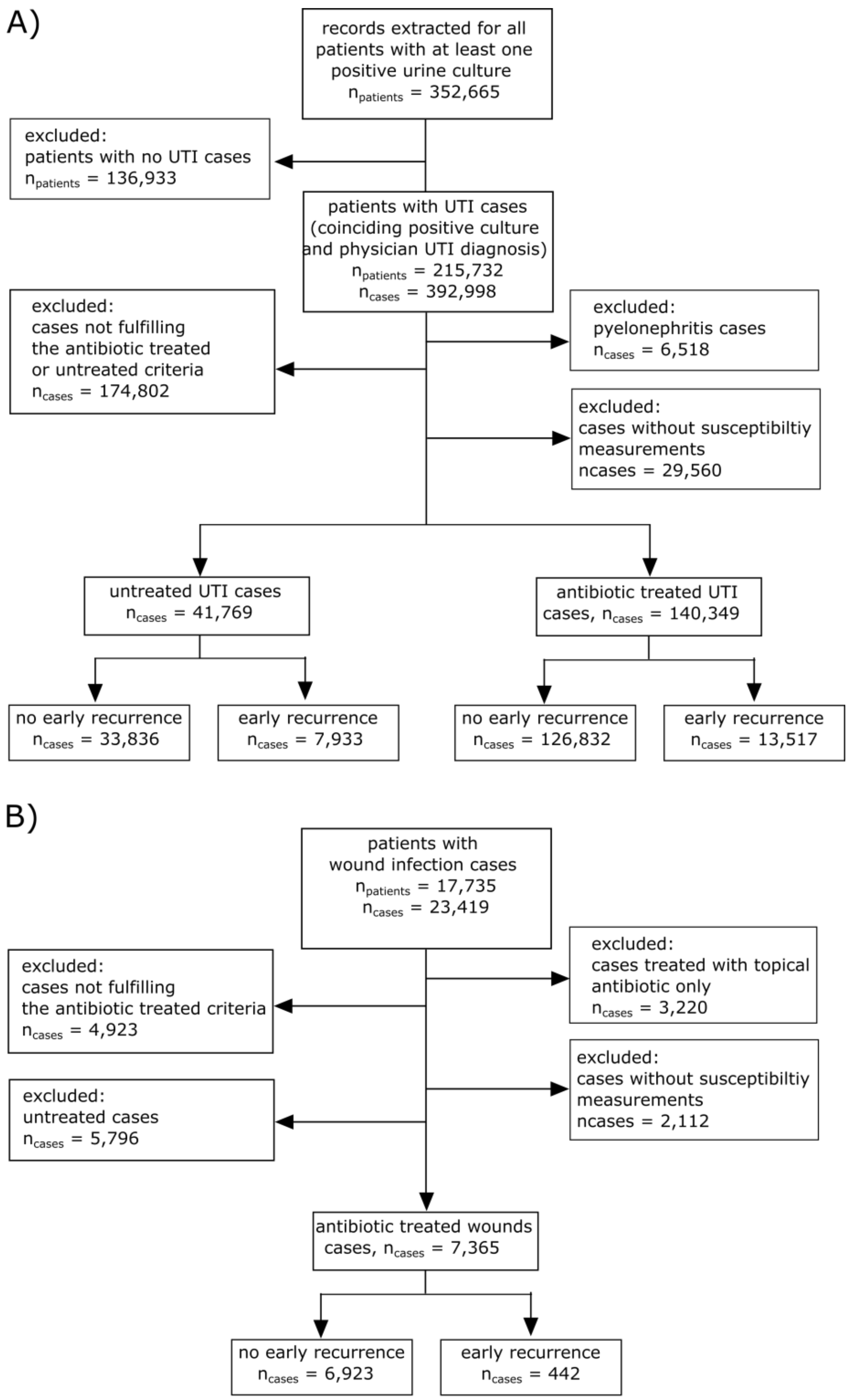

Figure S1. Flow diagram of the study design. A) Flow diagram indicating numbers of UTI patients and numbers of UTI cases included in the study. B) Flow diagram indicating numbers of wound infection patients and numbers of wound infection cases included in the study.

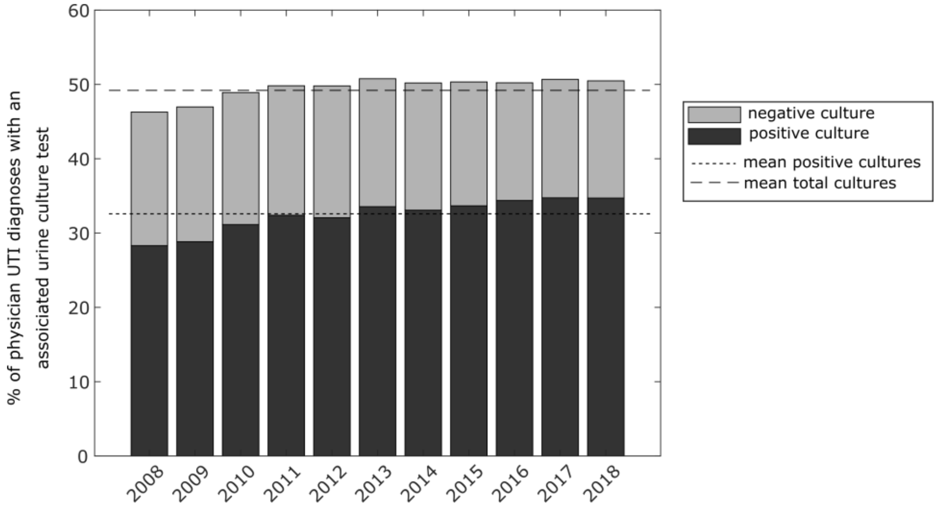

Fig. S2. The percentage of UTI diagnoses recorded each year which were associated with a urine culture. The percentage of physician UTI diagnoses records for which a urine culture for that patient, either negative or positive, was taken within seven days of the diagnosis.

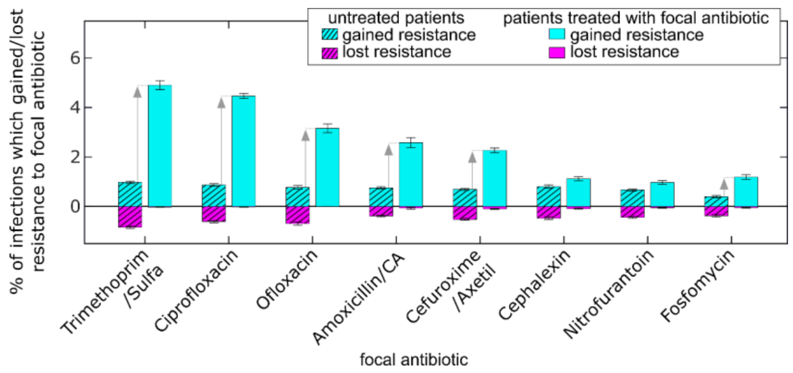

Fig. S3. Net change in resistance in antibiotic treated and untreated UTIs. For all antibiotic treated UTIs (solid bars), the risk of early recurrence with gained resistance (cyan, shown above the zero line) was significantly higher than the risk of early recurrence with loss of resistance (i.e. was initially resistant and recurred sensitive, magenta, shown below the zero line). Such bias towards gain of resistance was not seen in untreated infections (hashed bars), with <1% of cases which gained resistance matched by a similar fraction which lost resistance.

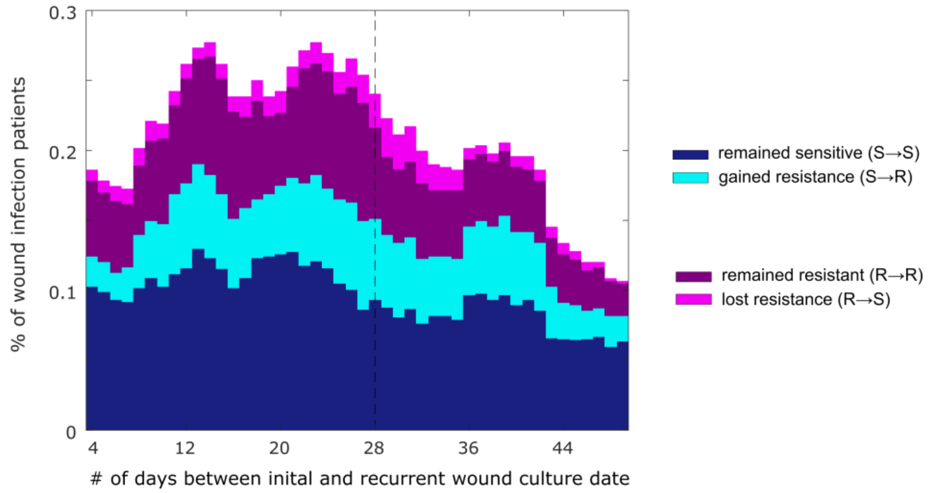

Fig. S4. Rate of wound infection recurrence by number days after treatment. The fraction of antibiotic treated wound infections which recurred on each day following the initial sample (7-day moving average). Each recurrent case is categorized by pre- and post- treatment susceptibility to the prescribed antibiotic

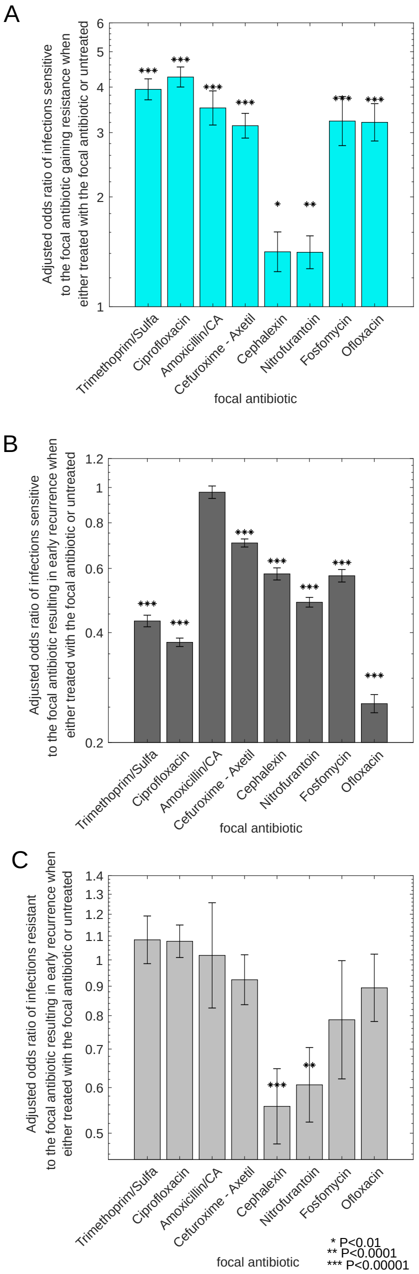

Fig. S5. Demographics adjusted risk of UTI recurrence and emergence of resistance with and without antibiotic treatment. A) Odds ratio adjusted for demographics (age, gender, pregnancy, previous catheter use) of early recurrence (within 4-28 days after the first sample) which gained resistance to the focal antibiotic. For each focal antibiotic the odds ratio compares cases initially sensitive to the focal antibiotic which are either treated with the focal antibiotic or untreated. B) Adjusted odds ratio of early recurrence (including recurrences which gained resistance and those which remained sensitive). For each focal antibiotic the odds ratio compares cases initially sensitive to the focal antibiotic which are either treated with the focal antibiotic or untreated. C) Adjusted odds ratio of early recurrence for infections initially resistant to the focal antibiotic which are either treated with the focal antibiotic or untreated.

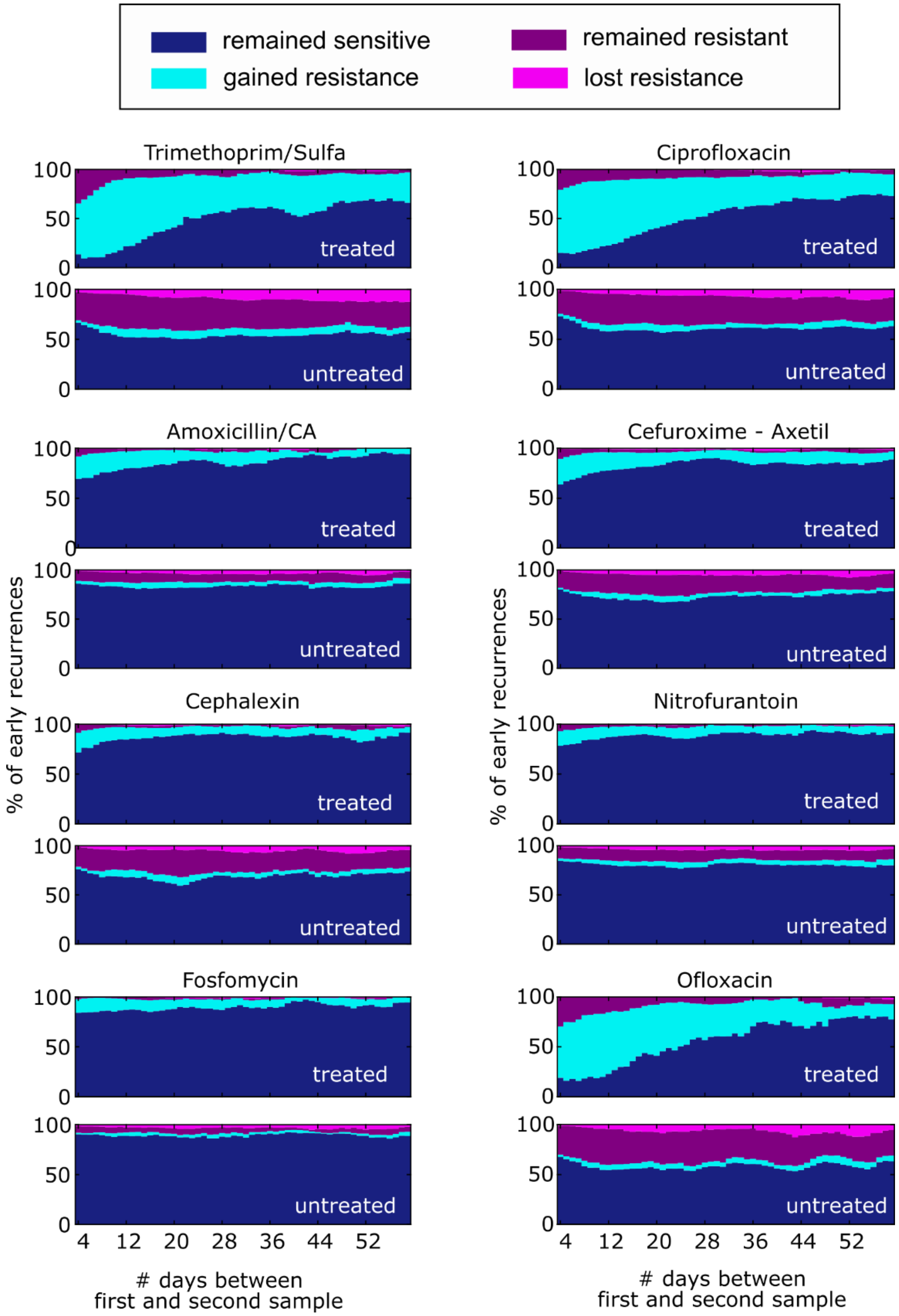

Fig. S6. Mode of recurrence by day for treated and untreated UTIs. The percentage of recurrent cases categorized by the initial and recurrent sample susceptibility to the specified antibiotic which recurred on each day between 4-60 days after the first sample (seven-day moving average). For each specified antibiotic the top plot shows cases treated with that antibiotic and the bottom plot shows untreated cases.

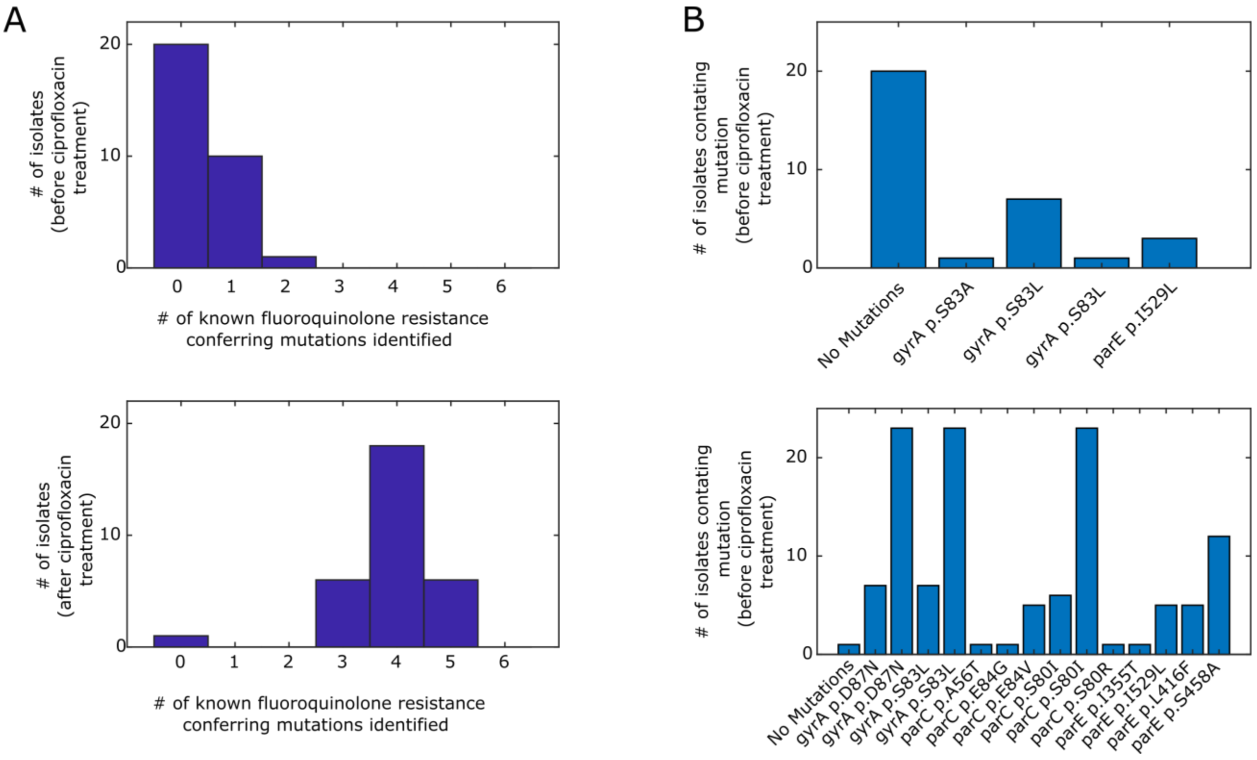

Fig. S7. Genetic and phenotypic fluoroquinolone resistance in sequenced UTI isolates. A) The number fluoroquinolone resistance conferring SNPs identified in sequenced isolates from ciprofloxacin treated cases which recurred with gained resistance to ciprofloxacin. The top panel shows isolates from samples before ciprofloxacin treatment and the bottom panel show isolates from infections after ciprofloxacin treatment. B) The number of sequenced isolates carrying specific mutations for ciprofloxacin treated cases which gained resistance to ciprofloxacin separated into before treatment (top) and after treatment (bottom).

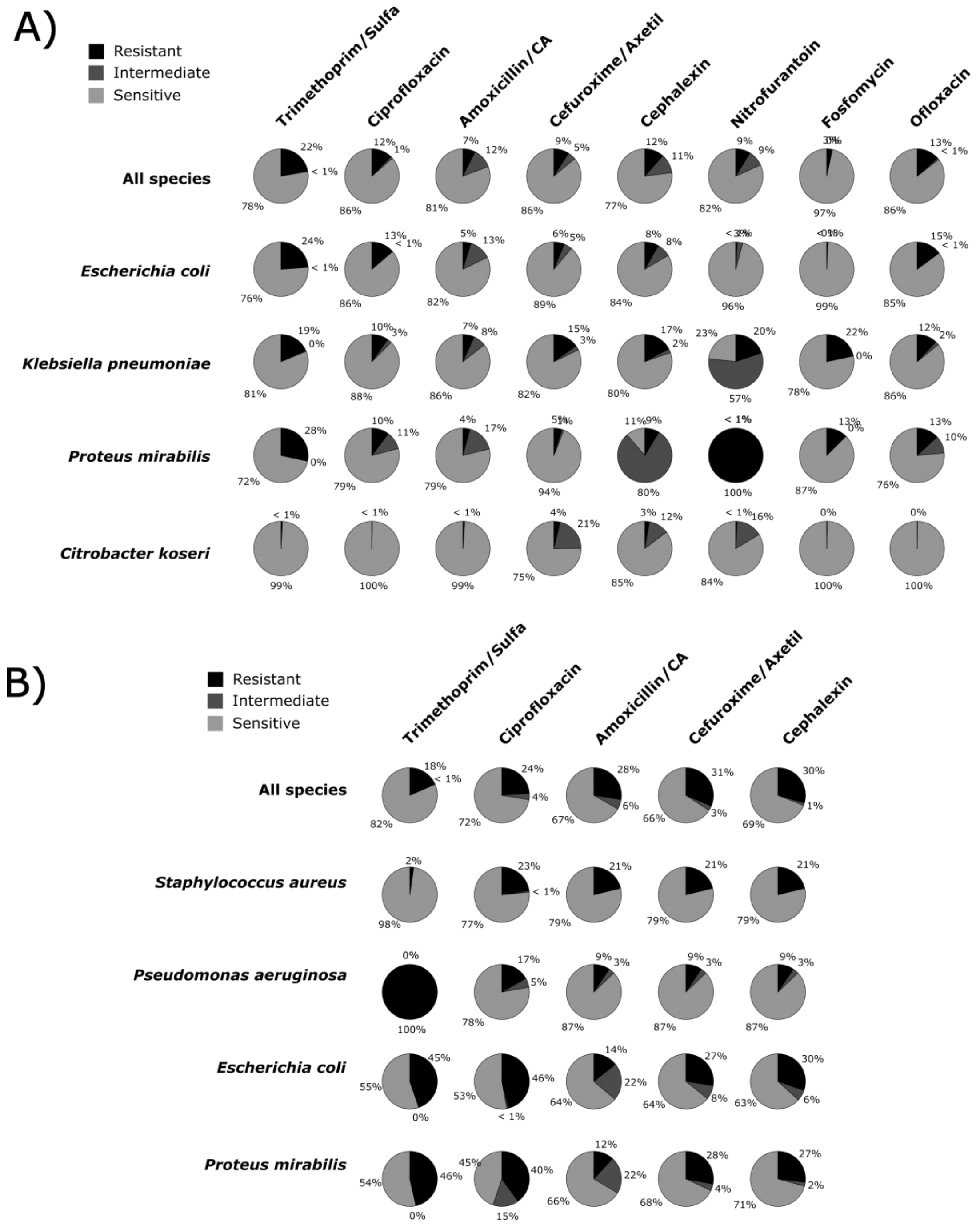

Fig. S8. Rates of resistance by species. A) Percentage of UTI isolates which are resistant, intermediate, and sensitive for each of the eight most commonly prescribed antibiotics. Average resistance rates are shown for all species (top), and for isolates identified as one of the four most common species. The percentage of all isolates identified as each species is shown in brackets. B) Percentage of wound infection isolates which are resistant, intermediate, and sensitive for each of the five most commonly prescribed antibiotics shown for the all species together (top) and the four most common species.

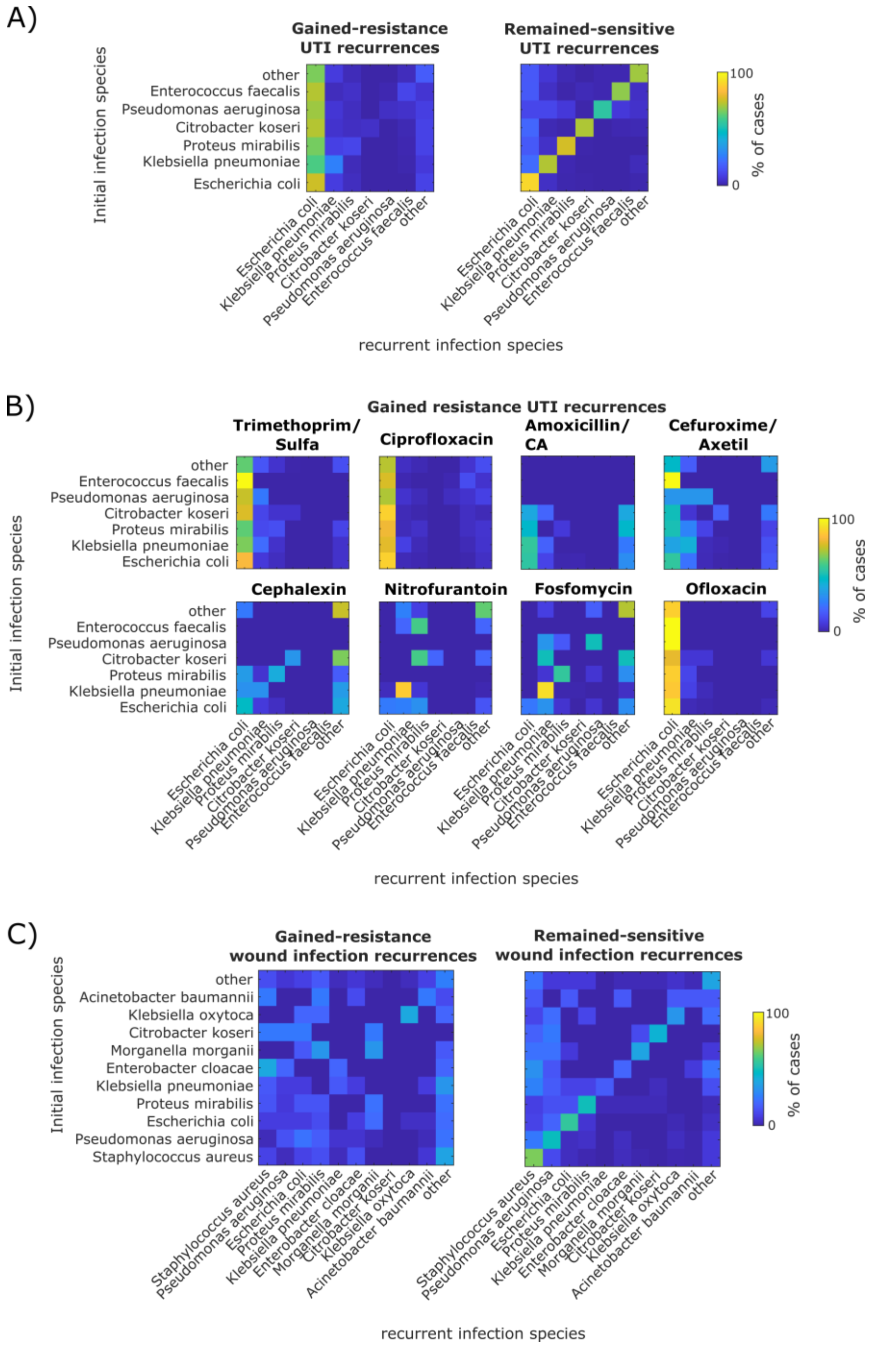

Fig. S9. Rates of species change for early recurrent cases. A) Plots showing the change of species for UTIs which recurred following any susceptibility-matched antibiotic treatment, where the recurrent infection either gained resistance to the treated antibiotic (left) or remained sensitive (right). Each row represents cases where the initial infection was caused by the specified species (y-axis) and shows the percentage of post-treatment recurrent infections caused by each species (x-axis). B) Plots of change of species in gained-resistance UTI recurrences for each antibiotic separately. C) Plots showing the change of species for wound infections which recurred following any susceptibility-matched antibiotic treatment, where the recurrent infection either gained resistance to the treated antibiotic (left) or remained sensitive (right).

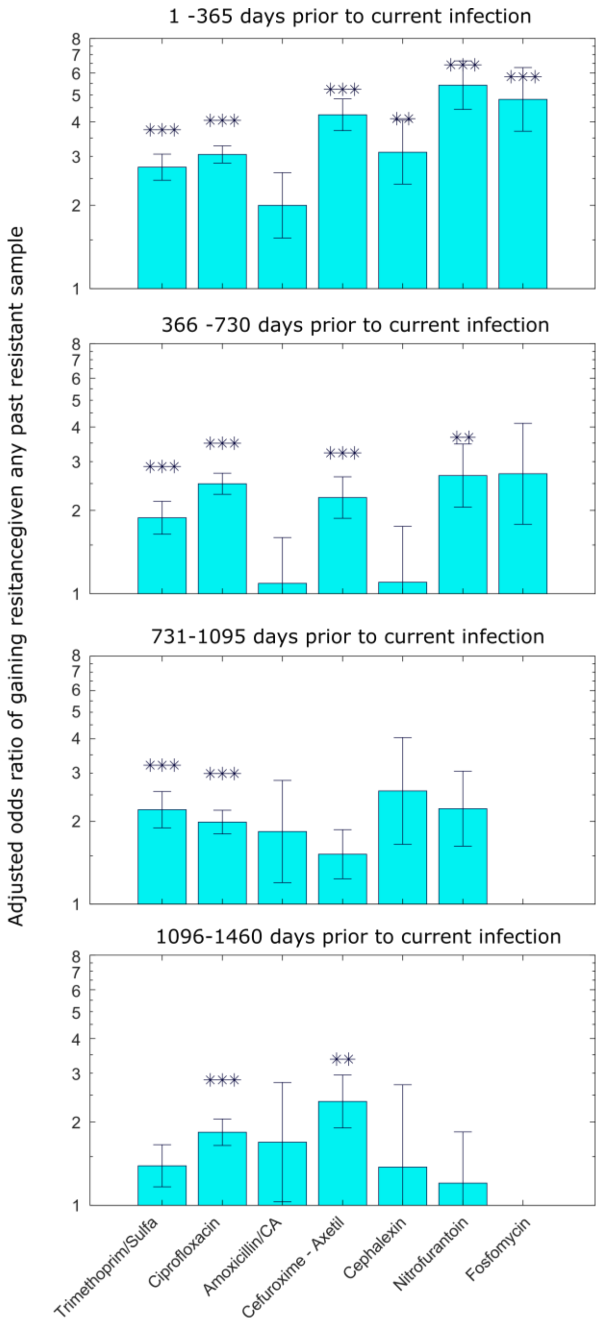

Fig. S10. Association of risk or emergence of resistance with past UTI susceptibility by year of prior sample. Odds ratio adjusted for demographics (age, gender, pregnancy, previous catheter use) of early recurrence which gained resistance following treatment for patients with any prior samples resistant to the treatment antibiotic compared to those with only prior samples susceptible to the treatment antibiotic during the one year period. Each plot shows the odds ratio when past samples for each one-year period prior to the current infection are considered.

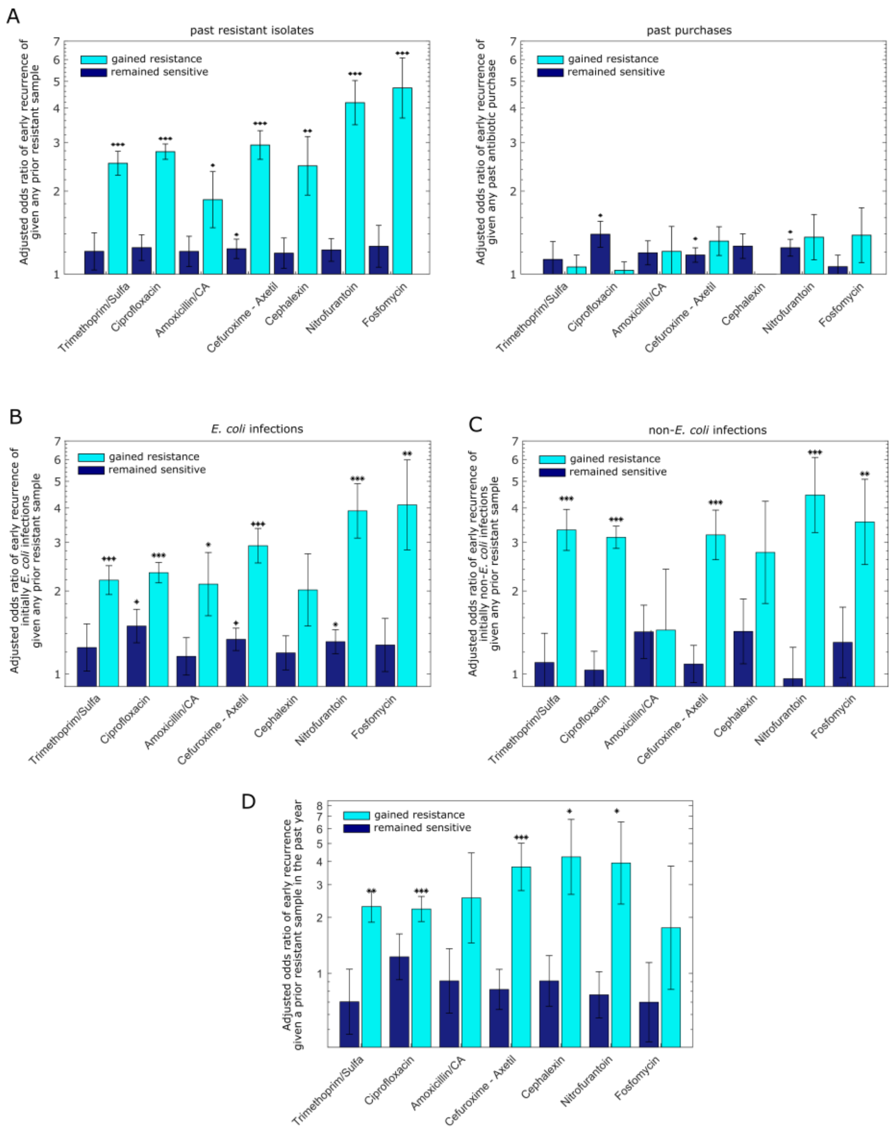

Fig. S11. Association of past antibiotic purchases compared to past UTI susceptibility with risk or emergence of resistance. A) Regression models for each treatment antibiotic as presented in Fig. 3B, but also accounting for prior purchase of that antibiotic as well as prior resistant isolates to that antibiotic and patient demographics. The odds ratio is shown for early recurrences following susceptibility-matched treatment which remained sensitive (blue) and those that gained resistance to the treatment antibiotic (cyan). The left panel shows the odds ratio of early recurrence given any prior resistant isolate adjusted for demographics and any prior purchases. The right panel shows the odds ratio of early recurrence given any prior purchase of the treatment antibiotic adjusted for and any prior resistant isolate. B) Adjusted odds ratio of early recurrence following susceptibility-matched treatment as presented in Fig. 3B but for only initially *E. coli* cases. C) Adjusted odds ratio of early recurrence following susceptibility-matched treatment as presented in Fig. 3B but for only initially non-*E. coli* cases. D) Adjusted odds ratio of early recurrence following susceptibility-matched treatment for patients with exactly one prior sample in the previous year. Patients whose prior sample was resistant to the treatment antibiotic are compared to those whose prior sample was susceptible to the treatment antibiotic. * $p < 0.05$; ** $p < 0.005$; *** $p < 0.0005$.

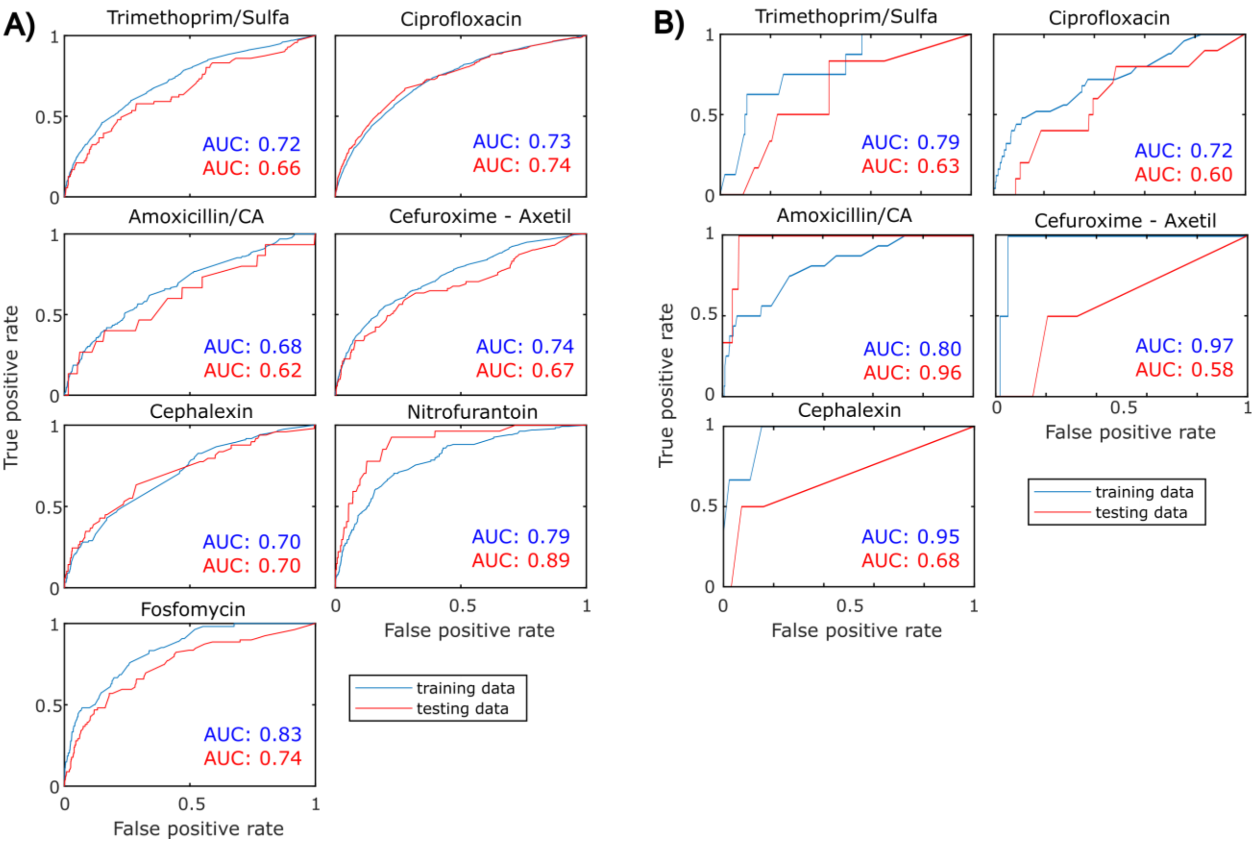

Fig. S12. Receiver operating characteristics for ML models. For each antibiotic the ROC curves shown for the logistic regression models for UTIs (A) and wound infections (B). The ROC curves and AUCs are shown for the temporally separated training data (blue) and the testing data (red).

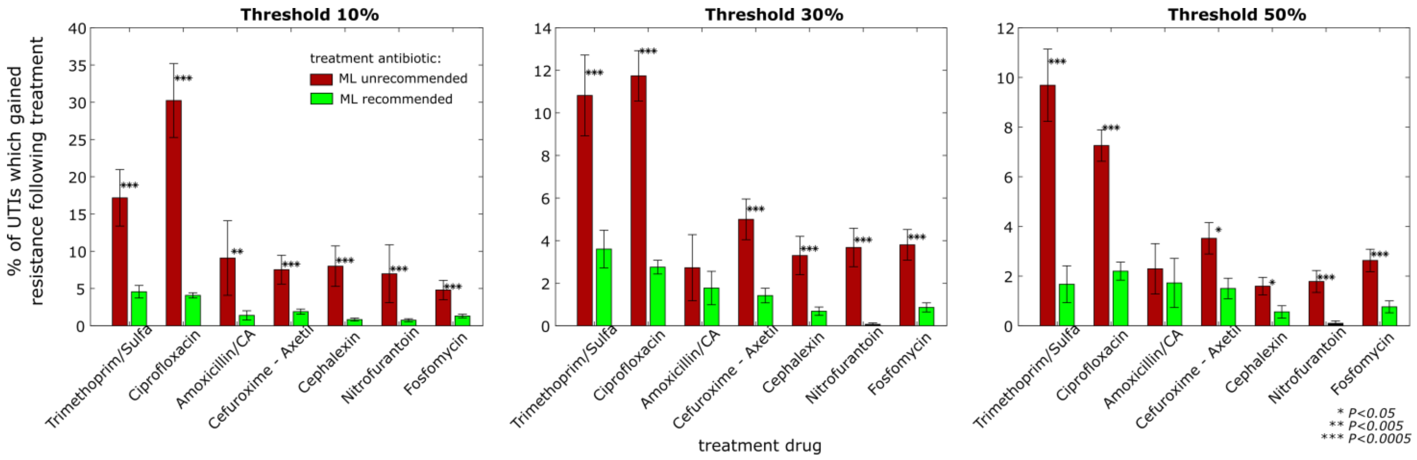

Fig. S13. Percentage of UTIs which gained resistance following treatment with unrecommended or recommended antibiotics categorized using three different binarization thresholds. Analysis as presented in Fig. 3E showing the percentage of UTIs within the test period which gained resistance following treatment. Cases were categorized into unrecommended (red) or recommended (green) by binarizing the ML algorithm predicted risk. The left panel shows a threshold of 10% highest risk to 90% lowest risk; middle panel: 30% highest risk to 70% lowest risk; right panel: 50% highest risk to 50% lowest risk.

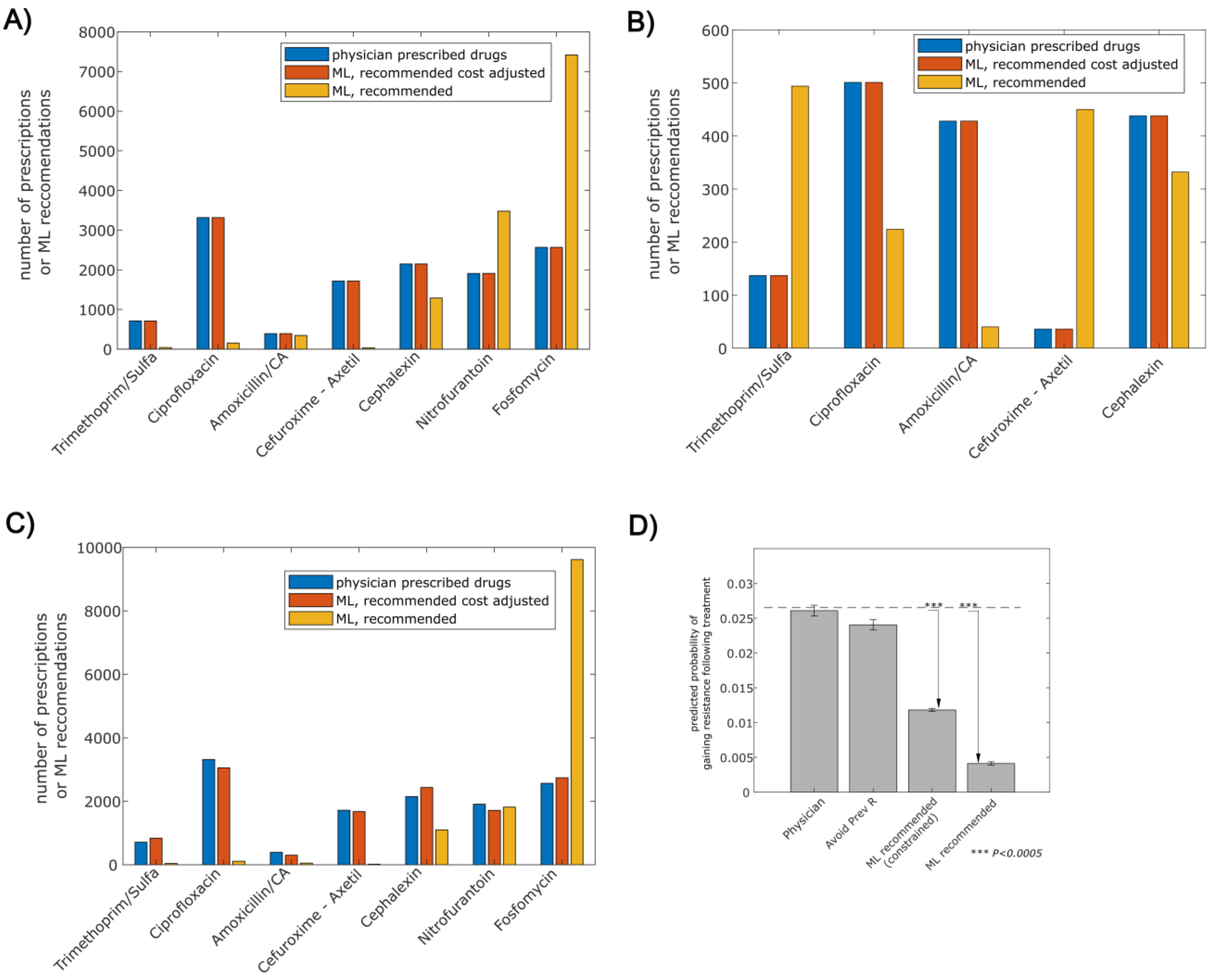

Fig. S14. Number of prescribed and machine learning recommended antibiotics. A) The distribution of antibiotics prescribed by physicians (blue bars), by the unconstrained ML algorithm (yellow bars), and constrained ML algorithm (red bars) for all UTI cases analyzed during the 14-month test period. B) The distribution of prescribed and ML recommended antibiotics for all wound infection cases analyzed during the 30-month test period. C) The distribution of prescribed and ML recommended antibiotics for all UTI cases showing the performance of the algorithm when the ML models were trained over one period (June 2007 to December 2016), cost adjusted in a second period (January 2017 to January 2018), and evaluated in a third period (February 2017 to January 2019). D) The overall predicted probability of gaining resistance for all UTIs during the final evaluation period (February 2017 to January 2019) as described above. The probability is shown for 4 different antibiotic prescription methods: the actual antibiotic prescribed by the physician; an algorithm that randomly chooses an antibiotic but avoids antibiotics to which the patient had past resistance, and the ML recommendation either unconstrained, or constrained such that each antibiotic is recommended at the same frequencies as prescribed by the physicians during the cost adjustment period (January 2017 to January 2018). * $p < 0.05$; ** $p < 0.005$; *** $p < 0.0005$.

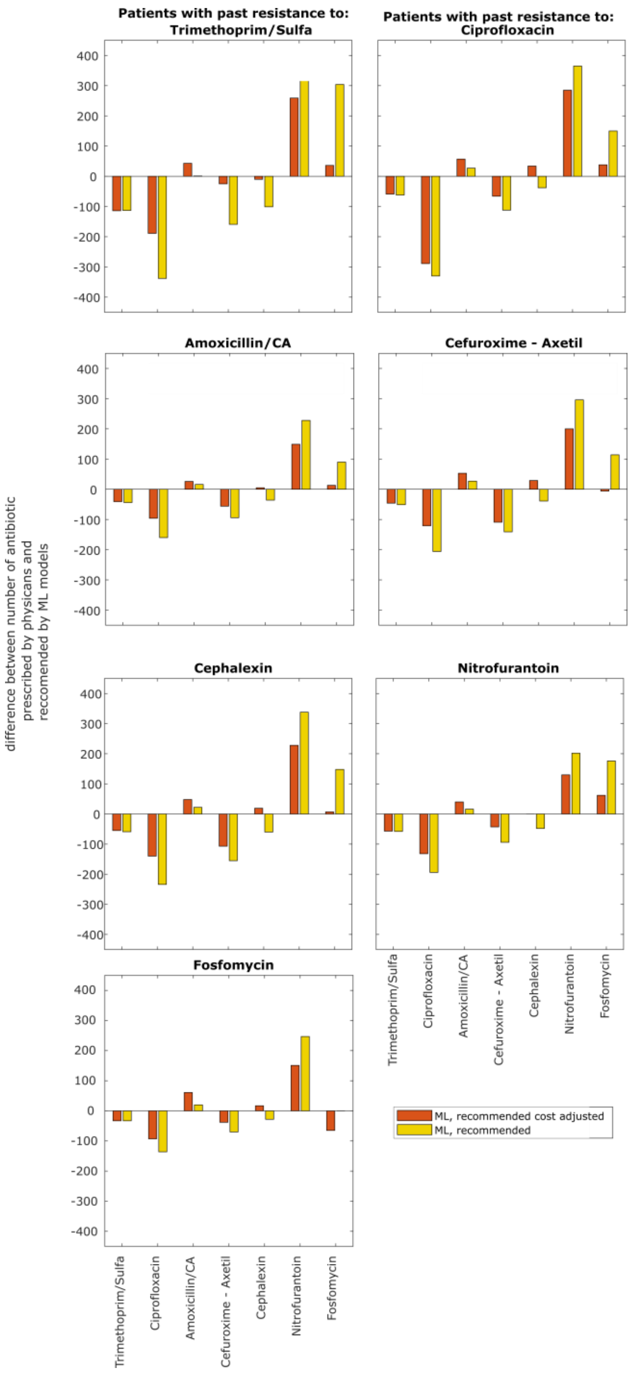

Fig. S15. Difference between the number of UTI antibiotics prescribed by physicians and recommended by the ML algorithm for patients with past isolates resistance to each antibiotic. The difference between the number of antibiotics prescribed by physicians and recommended by the ML algorithm, either unconstrained (yellow bars), or constrained (red bars), for all UTIs from the subset of patients whose current infection is sensitive to the specified antibiotic, but who have prior records of UTIs resistant to that antibiotic.

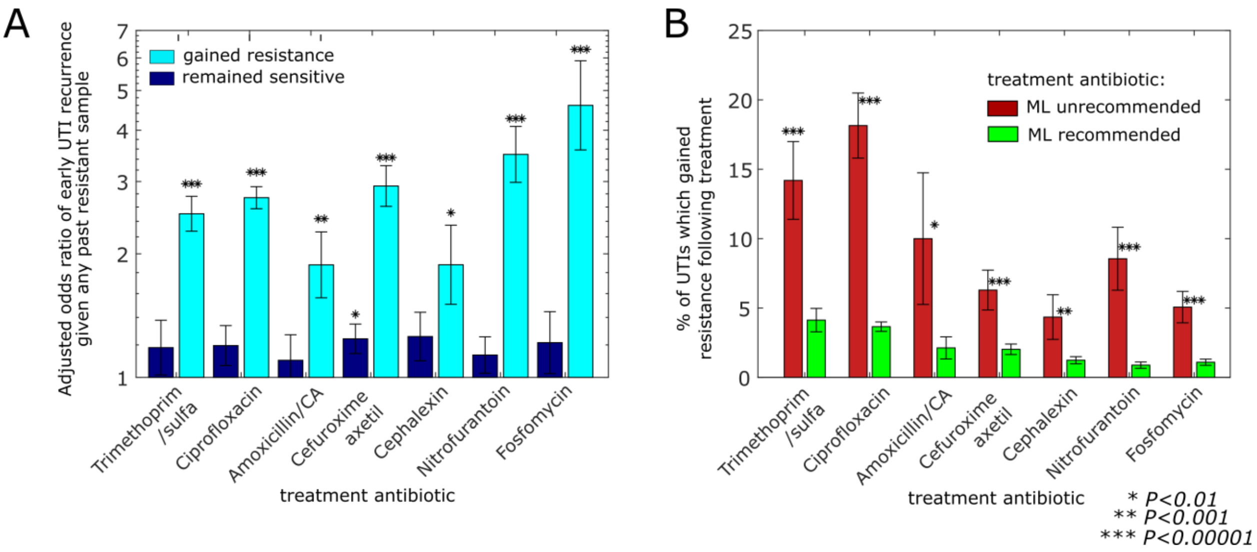

Fig. S16. Predictive model for gain of resistance with intermediate resistance grouped with resistant. A) Analysis as presented in Fig. 3B with intermediate resistant infections grouped with resistant. Odds ratio adjusted for demographics (age, gender, pregnancy, previous catheter use) of recurrence within 4-28 days following susceptibility-matched treatment for patients with any prior sample was resistant to the treatment antibiotic compared to those whose prior sample was susceptible to the treatment antibiotic. The odds ratio is shown for early recurrences which remained sensitive (blue) and those that gained resistance to the treatment antibiotic (cyan). B) Analysis as presented in Fig. 3E with intermediate resistant infections grouped with resistant. The percentage of UTIs within the 14-month test period which gained resistance following treatment for cases prescribed an antibiotic that was unrecommended (red, 15% highest predicted risk) or recommended (green, 85% lowest predicted risk) by the ML algorithm.

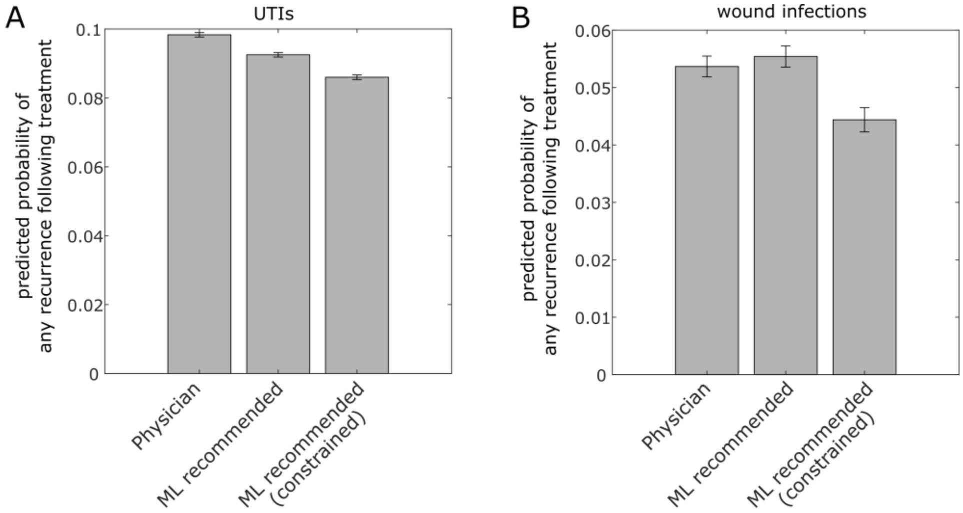

Fig. S17. The effect of minimization of gain of resistance by ML antibiotic recommendations on the overall risk of recurrence. The overall predicted probability of any recurrence for UTIs (A) and wounds (B) during the test period for 3 different antibiotic prescription methods: the actual antibiotic prescribed by the physician, and the ML antibiotic recommendation which best reduce risk of gain of resistance, either unconstrained, or constrained such that each antibiotic is recommended at the exact same frequencies as prescribed by the physicians.

Table S1. UTI case demographics						
	**All treated cases**	**Treated early recurrent cases**	**Treated early recurrent cases with sequenced isolates**	**All untreated cases**	**Untreated early recurrent cases**	**Untreated early recurrent cases with sequenced isolates**
Female – no. (%)	127506 (91)	11875 (88)	361 (90)	36795 (88)	6709 (85)	174 (88)
Male – no. (%)	12843 (9)	1642 (12)	40 (10)	4974 (12)	1224 (15)	23 (12)
Pregnant – no. (%)	4948 (4)	730 (5)	37 (9)	2700 (6)	653 (8)	18 (9)
Age – no. (%)						
0-9	10883 (8)	1177 (9)	34 (8)	5343 (13)	1082 (14)	30 (15)
10-19	7530 (5)	597 (4)	21 (5)	2747 (7)	468 (6)	13 (7)
20-29	18602 (13)	1190 (9)	41 (10)	5280 (13)	719 (9)	24 (12)
30-39	20070 (14)	1436 (11)	45 (11)	5633 (13)	888 (11)	20 (10)
40-49	21265 (15)	1352 (10)	40 (10)	4644 (11)	695 (9)	12 (6)
50-59	17977 (13)	1455 (11)	45 (11)	3976 (10)	719 (9)	9 (5)
60-69	19455 (14)	2294 (17)	55 (14)	4738 (11)	1074 (14)	34 (17)
70-79	15138 (11)	2328 (17)	80 (20)	5062 (12)	1210 (15)	30 (15)
80-89	7990 (6)	1423 (10)	36 (9)	3524 (8)	882 (11)	23 (12)
>90	1439 (1)	265 (2)	4 (1)	822 (2)	196 (2)	2 (1)
Any history of urinary catheterization	4305 (3)	817 (6)	18 (4)	2458 (6)	696 (9)	14 (7)
Recent urinary catheter use†	1321 (1)	238 (2)	6 (1)	776 (2)	225 (3)	2 (1)
Chronic Kidney Disease	15828 (11)	2674 (20)	90 (22)	6216 (15)	1550 (20)	42 (21)
Diabetes	14776 (11)	2154 (16)	61 (15)	5086 (12)	1220 (15)	31 (16)
Previous number of positive urine cultures* – no. (%)						
0-1	100448 (72)	6999 (52)	206 (51)	24582 (59)	3678 (46)	83 (42)
2-4	27241 (19)	3470 (26)	94 (23)	9449 (23)	1999 (25)	51 (26)
5+	12660 (9)	3048 (23)	101 (25)	7738 (19)	2256 (28)	63 (32)

*Number of previous positive urine cultures the patient has experienced within the past 3 years. †Catheter use within the past 30 days.

Table S2. Wound infection case demographics		
	**All treated cases**	**Treated early recurrent cases**
Female – no. (%)	3329 (45)	197 (45)
Male – no. (%)	4036 (55)	245 (55)
Pregnant – no. (%)	9 (0)	0 (0)
Age – no. (%)		
0-39	2205 (30)	33 (7)
40-49	874 (12)	52 (12)
50-59	1090 (15)	90 (20)
60-69	1267 (17)	105 (24)
70-79	1016 (14)	92 (21)
80-89	706 (10)	50 (11)
>90	207 (3)	20 (5)
Previous number of positive cultures* – no. (%)		
0-1	6395 (87)	288 (65)
2-4	636 (9)	88 (20)
5+	334 (5)	66 (15)

*Number of previous positive wound cultures the patient has experienced within the past 3 years.

**Table S3. Typical antibiotic dosing**		
**Antibiotic**	**Days**	**Dose**
Trimethoprim-sulfa	Twice-daily 5 days	160/800 mg
Ciprofloxacin	Twice-daily 5 days	250 mg
Amoxicillin/CA	Twice-daily 7 days	500 mg
Cefalexin	Twice-daily 5 days	500 mg
Cefuroxime	Twice-daily 7 days	250 mg
Nitrofurantoin	Four times per day 5 days	50 mg
Fosfomycin	Single	3-g
Ofloxacin	Twice-daily 10 days	250 mg

**Table S4. Species prevalence**			
**UTIs**		**Wound infections**	
**Species**	**%**	**Species**	**%**
Escherichia coli	75	*Staphylococcus aureus*	36
Klebsiella pneumoniae	9	*Pseudomonas aeruginosa*	13
Proteus mirabilis	5	*Escherichia coli*	9
Citrobacter koseri	3	*Proteus mirabilis*	7
Enterococcus faecalis	2	*Klebsiella pneumoniae*	6
Pseudomonas aeruginosa	1	*Enterobacter cloacae*	4
Enterobacter aerogenes	1	*Acinetobacter baumannii*	3
Enterobacter cloacae	1	*Citrobacter koseri*	3
Staphylococcus saphrophyticus	1	*Morganella morganii*	2
Staphylococcus aureus	1	*Klebsiella oxytoca*	2
Staphylococcus epidermidis	0	*Acinetobacter lwoffii*	2

Table S5. The fraction of sequenced recurrent *E . coli* UTI cases caused by reinfection with a different *E . coli* strain*

Treatment Antibiotic	All Cases	Gained Resistance	Remained Sensitive	Lost Resistance	Remained Resistant
Trimethoprim/Sulfa	12/23	9/12	1/6	0/0	2/5
Ciprofloxacin	41/71	31/31	6/25	1/1	3/14
Amoxicillin/CA	10/31	2/3	8/28	0/0	0/0
Cefuroxime/Axetil	23/76	6/6	17/67	0/0	0/3
Cephalexin	9/54	1/1	8/53	0/0	0/0
Nitrofurantoin	12/66	0/0	12/66	0/0	0/0
Fosfomycin	7 /74	0/0	7 /74	0/0	0/0
Ofloxacin†	3 /6	2 /2	1 /3	0 /0	0 /1
Untreated	26/197				

*number of cases caused by a different strain / total number of cases.

†Susceptibility to ciprofloxacin was used as a proxy for ofloxacin since ofloxacin susceptibility was not routinely measured during the collection period.

Table S6. Resistance genes identified in pre- and post-treatment UTI isolates in cases which gained resistance to the treatment antibiotic.

	**Pre-treatment isolate**			**No. of SNV difference**	**Post-treatment isolate**		
Treatment antibiotic	**β-lactam**	**Trimethoprim**	**Fluoroquinolones**		**β-lactam**	**Trimethoprim**	**Fluoroquinolones**
**cefuroxime**				10847	blaSHV-12; blaTEM-1B	dfrA1	
**cefuroxime**				6226	blaCTX-M-27		
**cefuroxime**				7354	blaCTX-M-27		
**cefuroxime**				1474	blaCTX-M-15; blaOXA-1	dfrA17	
**cefuroxime**				22058	blaCTX-M-27		
**cefuroxime**				14518	blaCTX-M-15; blaTEM-1B	dfrA14	
**ciprofloxacin**				13871			
**ciprofloxacin**				15131	blaTEM-1B		
**ciprofloxacin**	blaTEM-1B			28885		dfrA12	qnrS1
**ciprofloxacin**	blaCTX-M-15	dfrA17		14508		dfrA17	
**ciprofloxacin**	blaTEM-1B			344	blaTEM-1B		
**ciprofloxacin**	blaTEM-57			6631	blaTEM-1B	dfrA12	
**ciprofloxacin**				20456	blaCMY-2; blaCTX-M-15		
**ciprofloxacin**				13547	blaCTX-M-15	blaOXA-1	
**ciprofloxacin**	blaCTX-M-14b; blaTEM-1B	dfrA1	qnrB4	32180			
**ciprofloxacin**	blaTEM-1B			18996			
**ciprofloxacin**				31819	blaTEM-1B	dfrA1	
**ciprofloxacin**				22774	blaTEM-1B	dfrA14	
**ciprofloxacin**				21573	blaCTX-M-15; blaOXA-1	dfrA17	
**ciprofloxacin**	blaCMY-2; blaTEM-1A		qnrS1	30031	blaCFE-1; blaSHV-48		qnrB28
**ciprofloxacin**	blaCTX-M-24; blaTEM-1B	dfrA17		20668	blaTEM-1B	dfrA1; dfrA12	
**ciprofloxacin**	blaTEM-1B	dfrA14; dfrA5		7828	blaTEM-1B		
**ciprofloxacin**				14777	blaTEM-1B	dfrA1	
**ciprofloxacin**		dfrA14		11409	blaCTX-M-15; blaOXA-1	dfrA14	
**ciprofloxacin**	blaTEM-1B	dfrA17		14829	blaCTX-M-15; blaTEM-1B	dfrA17	
**ciprofloxacin**				6303	blaCTX-M-14		
**ciprofloxacin**		dfrA17		24829	blaCTX-M-27	dfrA17	
**ciprofloxacin**	blaTEM-1B			13993	blaTEM-1B		
**ciprofloxacin**	blaCTX-M-15; blaTEM-1B	dfrA17		13792			
**ciprofloxacin**				33480		dfrA12	
**ciprofloxacin**				26154	blaTEM-1B	dfrA12	
**ciprofloxacin**				24734	blaTEM-1B		
**ciprofloxacin**	blaSHV-48			16075	blaCMY-2		
**ciprofloxacin**				12028	blaTEM-1C		
**ciprofloxacin**	blaTEM-1B	dfrA17		32101	blaCTX-M-27	dfrA17	
**ciprofloxacin**	blaTEM-1B			14743	blaCTX-M-15; blaTEM-1B	dfrA1	qnrS1
**ciprofloxacin**	blaTEM-1B			7192	blaTEM-1B		
**trimethoprim/sulfa**	blaTEM-1B	dfrA1		17176	blaTEM-1B	dfrA1	
**trimethoprim/sulfa**				14900	blaCTX-M-15; blaTEM-1B	dfrA14	qnrS1
**trimethoprim/sulfa**				9505		dfrA14	
**trimethoprim/sulfa**	blaCTX-M-15; blaOXA-1			4548		dfrA14	
**trimethoprim/sulfa**				2681	blaTEM-1B	dfrA7	
**trimethoprim/sulfa**	blaTEM-1B			26722		dfrA17	
**trimethoprim/sulfa**				14518	blaCTX-M-15; blaTEM-1B	dfrA14	
**trimethoprim/sulfa**				24558		dfrA7	
**trimethoprim/sulfa**				19553	blaCTX-M-27	dfrA17	
**trimethoprim/sulfa**				12	blaTEM-1B	dfrA17	
**trimethoprim/sulfa**	blaTEM-1B			13	blaTEM-1B	dfrA17	
**trimethoprim/sulfa**	blaCTX-M-15; blaTEM-1B			0	blaCTX-M-15; blaTEM-1B	dfrA17	
**amoxicillin/ca**	blaTEM-1B	dfrA14		23906	blaCMY-2		
**amoxicillin/ca**	blaTEM-1B		qnrS1	460	blaTEM-57	dfrA14	qnrS1
**amoxicillin/ca**				3	blaTEM-1B	dfrA14	
**cefalexin**				19553	blaCTX-M-27	dfrA17	

Table S7. Regression coefficients for the risk of early recurrence which gained resistance following sensitivity-matched treatment given any past sample resistance to the treatment antibiotic.

	UTI: Trimethoprim/Sulfa	*UTI: Ciprofloxacin*
Age 0-9	*0.37 [0.15, 0.59]*	*NaN [NaN, NaN]*
Age 10-19	*0.18 [-0.09, 0.45]*	*-1.30 [-2.31,-0.28]*
Age 20-29	*-0.37 [-0.64,-0.10]*	*-0.50 [-0.66,-0.34]**
Age 30-39	*0.08 [-0.15, 0.32]*	*-0.46 [-0.61,-0.31]**
Age 40-49	*-0.11 [-0.33, 0.12]*	*-0.53 [-0.67,-0.40]****
Age 60-69	*0.59 [0.40, 0.78]**	*0.35 [0.25, 0.46]***
Age 70-79	*0.81 [0.62, 0.99]****	*0.57 [0.47, 0.67]****
Age 80-89	*0.63 [0.42, 0.84]**	*0.77 [0.66, 0.88]****
Age 90-100	*0.62 [0.27, 0.97]*	*0.71 [0.51, 0.91]***
Gender	*0.08 [-0.10, 0.26]*	*0.42 [0.32, 0.52]****
Pregnancy	*0.55 [-0.07, 1.16]*	*0.93 [0.46, 1.41]*
Catheter	*0.32 [0.13, 0.52]*	*0.40 [0.28, 0.52]***
Prev Resistance	*0.92 [0.82, 1.02]****	*1.00 [0.94, 1.06]****

	UTI: Amoxicillin/CA	*UTI: Cefuroxime - Axetil*
Age 0-9	*-0.27 [-0.70, 0.15]*	*-0.05 [-0.37, 0.28]*
Age 10-19	*0.03 [-0.47, 0.52]*	*-0.53 [-0.88,-0.19]*
Age 20-29	*-0.24 [-0.78, 0.31]*	*-0.45 [-0.74,-0.17]*
Age 30-39	*-0.60 [-1.14,-0.06]*	*-0.52 [-0.79,-0.25]*
Age 40-49	*-0.12 [-0.64, 0.40]*	*-0.13 [-0.38, 0.12]*
Age 60-69	*-0.63 [-1.18,-0.08]*	*0.53 [0.31, 0.75]*
Age 70-79	*0.52 [0.09, 0.96]*	*0.65 [0.43, 0.86]**
Age 80-89	*0.94 [0.50, 1.37]*	*0.74 [0.51, 0.97]**
Age 90-100	*0.53 [-0.17, 1.23]*	*0.72 [0.37, 1.08]*
Gender	*0.50 [0.11, 0.89]*	*-0.10 [-0.30, 0.09]*
Pregnancy	*0.92 [0.51, 1.33]*	*-0.26 [-0.53, 0.02]*
Catheter	*-0.08 [-0.45, 0.29]*	*0.01 [-0.24, 0.26]*
Prev Resistance	*0.65 [0.41, 0.88]**	*1.11 [1.00, 1.23]****

	UTI: Cephalexin	*UTI: Nitrofurantoin*
Age 0-9	*0.34 [-0.14, 0.83]*	*1.31 [0.23, 2.38]*
Age 10-19	*-1.18 [-1.92,-0.45]*	*-0.31 [-1.37, 0.74]*
Age 20-29	*0.07 [-0.55, 0.69]*	*-0.26 [-0.80, 0.28]*
Age 30-39	*-0.32 [-1.00, 0.37]*	*-0.84 [-1.46,-0.21]*
Age 40-49	*-0.18 [-0.82, 0.46]*	*-0.27 [-0.77, 0.23]*
Age 60-69	*0.72 [0.19, 1.26]*	*0.78 [0.41, 1.15]*
Age 70-79	*0.86 [0.33, 1.40]*	*0.97 [0.61, 1.33]**
Age 80-89	*0.90 [0.32, 1.48]*	*1.26 [0.88, 1.63]***
Age 90-100	*NaN [NaN, NaN]*	*1.15 [0.62, 1.69]*
Gender	*-0.99 [-1.35,-0.63]**	*-0.86 [-1.10,-0.61]***
Pregnancy	*-1.12 [-2.17,-0.07]*	*0.53 [-0.13, 1.19]*
Catheter	*-0.80 [-1.33,-0.28]*	*0.25 [-0.05, 0.56]*
Prev Resistance	*0.87 [0.63, 1.11]***	*1.45 [1.26, 1.63]****

	UTI: Fosfomycin
Age 0-9	*NaN [NaN, NaN]*
Age 10-19	*-0.71 [-1.76, 0.33]*
Age 20-29	*-1.67 [-2.36,-0.97]*
Age 30-39	*-1.51 [-2.16,-0.87]*
Age 40-49	*-0.52 [-0.97,-0.07]*
Age 60-69	*0.25 [-0.13, 0.63]*
Age 70-79	*0.41 [0.05, 0.78]*
Age 80-89	*0.58 [0.20, 0.96]*
Age 90-100	*1.03 [0.46, 1.59]*
Gender	*-0.29 [-0.68, 0.10]*
Pregnancy	*1.55 [0.93, 2.18]*
Catheter	*0.87 [0.55, 1.20]**
Prev Resistance	*1.53 [1.28, 1.78]****

**Wound infections**	**All antibiotics**
Age 0-39	0.01 [-0.68, 0.70]
Age 40-49	-0.03 [-0.65, 0.59]
Age 60-69	-0.47 [-0.99, 0.05]
Age 70-79	0.16 [-0.31, 0.62]
Age 80+	0.12 [-0.38, 0.63]
Gender	-0.18 [-0.51, 0.16]
Pregnancy	NaN [NaN, NaN]
Prev Resistance	1.38 [1.05, 1.72]***

* P<0.01 ** P<0.0001 *** P<0.000001. Brackets indicate the 95% confidence interval.

Table S8. Regression coefficients for the risk of early recurrence which remained sensitive following sensitivity-matched treatment given any past sample resistance to the treatment antibiotic.

	UTI: Trimethoprim/Sulfa	UTI: Ciprofloxacin
Age 0-9	-0.27 [-0.54, 0.01]	NaN [NaN, NaN]
Age 10-19	-1.55 [-2.08,-1.02]*	NaN [NaN, NaN]
Age 20-29	-1.13 [-1.51,-0.74]*	-0.58 [-0.80,-0.35]
Age 30-39	-1.13 [-1.49,-0.77]*	-0.28 [-0.47,-0.09]
Age 40-49	-0.36 [-0.62,-0.10]	-0.23 [-0.40,-0.07]
Age 60-69	-0.37 [-0.60,-0.13]	0.02 [-0.12, 0.17]
Age 70-79	-0.43 [-0.67,-0.19]	0.02 [-0.13, 0.17]
Age 80-89	-0.89 [-1.21,-0.57]*	-0.10 [-0.28, 0.08]
Age 90-100	-0.82 [-1.44,-0.20]	-1.31 [-1.90,-0.71]
Gender	-1.09 [-1.29,-0.90]***	-0.60 [-0.72,-0.48]***
Pregnancy	1.64 [1.01, 2.28]	0.92 [0.32, 1.52]
Catheter	0.85 [0.62, 1.09]**	0.51 [0.33, 0.68]*
Prev Resistance	0.17 [0.01, 0.32]	0.25 [0.15, 0.36]

	UTI: Amoxicillin/CA	UTI: Cefuroxime - Axetil
Age 0-9	-0.49 [-0.69,-0.29]	-0.02 [-0.18, 0.15]
Age 10-19	-0.02 [-0.26, 0.21]	-0.04 [-0.19, 0.11]
Age 20-29	-0.14 [-0.40, 0.11]	-0.21 [-0.34,-0.08]
Age 30-39	-0.25 [-0.49,-0.01]	-0.11 [-0.23, 0.01]
Age 40-49	-0.24 [-0.49, 0.01]	-0.11 [-0.24, 0.01]
Age 60-69	0.26 [0.04, 0.47]	0.30 [0.18, 0.42]
Age 70-79	0.43 [0.22, 0.65]	0.29 [0.17, 0.41]
Age 80-89	0.36 [0.14, 0.59]	0.33 [0.19, 0.46]
Age 90-100	0.58 [0.22, 0.93]	0.13 [-0.12, 0.37]
Gender	-0.36 [-0.52,-0.20]	-0.28 [-0.40,-0.16]
Pregnancy	0.31 [0.10, 0.52]	0.50 [0.40, 0.59]***
Catheter	-0.08 [-0.26, 0.10]	0.26 [0.11, 0.40]
Prev Resistance	0.21 [0.09, 0.34]	0.23 [0.15, 0.31]*

	UTI: Cephalexin	UTI: Nitrofurantoin
Age 0-9	-0.27 [-0.46,-0.09]	0.59 [0.09, 1.08]
Age 10-19	-0.42 [-0.63,-0.21]	0.05 [-0.21, 0.32]
Age 20-29	-0.57 [-0.81,-0.33]	-0.00 [-0.15, 0.15]
Age 30-39	-0.20 [-0.42, 0.03]	-0.25 [-0.41,-0.09]
Age 40-49	-0.35 [-0.58,-0.12]	0.01 [-0.13, 0.16]
Age 60-69	0.16 [-0.06, 0.37]	0.35 [0.22, 0.48]*
Age 70-79	0.22 [-0.01, 0.44]	0.38 [0.25, 0.51]*
Age 80-89	0.28 [0.02, 0.53]	0.60 [0.46, 0.75]***
Age 90-100	0.16 [-0.35, 0.68]	0.85 [0.62, 1.08]**
Gender	-0.38 [-0.59,-0.16]	-0.72 [-0.84,-0.60]***
Pregnancy	0.51 [0.30, 0.72]	0.69 [0.53, 0.85]***
Catheter	0.17 [-0.00, 0.35]	0.16 [-0.00, 0.32]
Prev Resistance	0.18 [0.06, 0.31]	0.22 [0.13, 0.31]

	UTI: Fosfomycin
Age 0-9	NaN [NaN, NaN]
Age 10-19	-0.58 [-0.99,-0.17]
Age 20-29	-0.47 [-0.69,-0.26]
Age 30-39	-0.10 [-0.30, 0.10]
Age 40-49	-0.53 [-0.73,-0.33]*
Age 60-69	0.36 [0.18, 0.53]
Age 70-79	0.61 [0.44, 0.78]**
Age 80-89	0.65 [0.46, 0.83]**
Age 90-100	-0.16 [-0.58, 0.27]
Gender	-0.61 [-0.81,-0.41]*
Pregnancy	0.91 [0.70, 1.12]***
Catheter	0.65 [0.46, 0.84]**
Prev Resistance	0.19 [0.02, 0.37]

**Wound infections**	**All antibiotics**
Age 0-39	-1.44 [-2.20,-0.67]
Age 40-49	0.53 [0.12, 0.93]
Age 60-69	0.58 [0.25, 0.91]
Age 70-79	0.16 [-0.20, 0.53]
Age 80+	0.35 [-0.03, 0.74]
Gender	0.03 [-0.20, 0.26]
Pregnancy	NaN [NaN, NaN]
Prev Resistance	-0.41 [-0.67,-0.16]

* P<0.01 ** P<0.0001 *** P<0.000001. Brackets indicate the 95% confidence interval.

References:

36. M. Baym, S. Kryazhimskiy, T. D. Lieberman, H. Chung, M. M. Desai, R. Kishony, Inexpensive Multiplexed Library Preparation for Megabase-Sized Genomes. PLoS One. 10, e0128036 (2015).

37. B. D. Ondov, T. J. Treangen, P. Melsted, A. B. Mallonee, N. H. Bergman, S. Koren, A. M. Phillippy, Mash: Fast genome and metagenome distance estimation using MinHash. Genome Biol. 17, 132 (2016).

38. A. Bankevich, S. Nurk, D. Antipov, A. A. Gurevich, M. Dvorkin, A. S. Kulikov, V. M. Lesin, S. I. Nikolenko, S. Pham, A. D. Prjibelski, A. V. Pyshkin, A. V. Sirotkin, N. Vyahhi, G. Tesler, M. A. Alekseyev, P. A. Pevzner, SPAdes: A new genome assembly algorithm and its applications to single-cell sequencing. J. Comput. Biol. 19, 455–477 (2012).
